# Differential Inhibition of APOBEC3 DNA‐Mutator Isozymes by Fluoro‐ and Non‐Fluoro‐Substituted 2′‐Deoxyzebularine Embedded in Single‐Stranded DNA

**DOI:** 10.1002/cbic.201900505

**Published:** 2019-12-19

**Authors:** Maksim V. Kvach, Fareeda M. Barzak, Stefan Harjes, Henry A. M. Schares, Harikrishnan M. Kurup, Katherine F. Jones, Lorraine Sutton, John Donahue, Richard T. D'Aquila, Geoffrey B. Jameson, Daniel A. Harki, Kurt L. Krause, Elena Harjes, Vyacheslav V. Filichev

**Affiliations:** ^1^ School of Fundamental Sciences Massey University Private Bag 11 222 Palmerston North 4442 New Zealand; ^2^ Maurice Wilkins Centre for Molecular Biodiscovery Private Bag 92019 Auckland 1142 New Zealand; ^3^ Department of Medicinal Chemistry University of Minnesota 2231 6th Street SE Minneapolis MN 55455 USA; ^4^ Department of Chemistry University of Minnesota 207 Pleasant St SE Minneapolis MN 55455 USA; ^5^ Department of Biochemistry University of Otago P. O. Box 56 Dunedin 9054 New Zealand; ^6^ Division of Infectious Diseases Department of Medicine Vanderbilt University School of Medicine 21st Ave S Nashville TN 37232 USA; ^7^ Division of Infectious Diseases and Northwestern HIV Translational Research Center Department of Medicine Northwestern University Feinberg School of Medicine 676 N. St. Clair Street Suite 2330 Chicago IL 60611 USA

**Keywords:** antitumor agents, APOBEC3, enzyme catalysis, fluorodeoxyzebularine, inhibitors

## Abstract

The APOBEC3 (APOBEC3A‐H) enzyme family is part of the human innate immune system that restricts pathogens by scrambling pathogenic single‐stranded (ss) DNA by deamination of cytosines to produce uracil residues. However, APOBEC3‐mediated mutagenesis of viral and cancer DNA promotes its evolution, thus enabling disease progression and the development of drug resistance. Therefore, APOBEC3 inhibition offers a new strategy to complement existing antiviral and anticancer therapies by making such therapies effective for longer periods of time, thereby preventing the emergence of drug resistance. Here, we have synthesised 2′‐deoxynucleoside forms of several known inhibitors of cytidine deaminase (CDA), incorporated them into oligodeoxynucleotides (oligos) in place of 2′‐deoxycytidine in the preferred substrates of APOBEC3A, APOBEC3B, and APOBEC3G, and evaluated their inhibitory potential against these enzymes. An oligo containing a 5‐fluoro‐2′‐deoxyzebularine (5FdZ) motif exhibited an inhibition constant against APOBEC3B 3.5 times better than that of the comparable 2′‐deoxyzebularine‐containing (dZ‐containing) oligo. A similar inhibition trend was observed for wild‐type APOBEC3A. In contrast, use of the 5FdZ motif in an oligo designed for APOBEC3G inhibition resulted in an inhibitor that was less potent than the dZ‐containing oligo both in the case of APOBEC3G_CTD_ and in that of full‐length wild‐type APOBEC3G.

## Graphical abstract

**Design of ssDNAs as inhibitors of A3 enzymes with the aid of chemically modified nucleosides**: Incorporation of 5‐fluoro‐2′‐deoxyzebularine into single‐stranded DNA in place of the target dC residue yielded more powerful inhibition of APOBEC3A and APOBEC3B—but not of full‐length APOBEC3G—than that observed with the non‐fluorinated 2′‐deoxyzebularine.

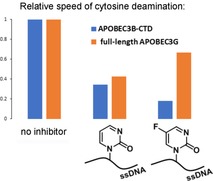

## Introduction

**Scheme 1:**
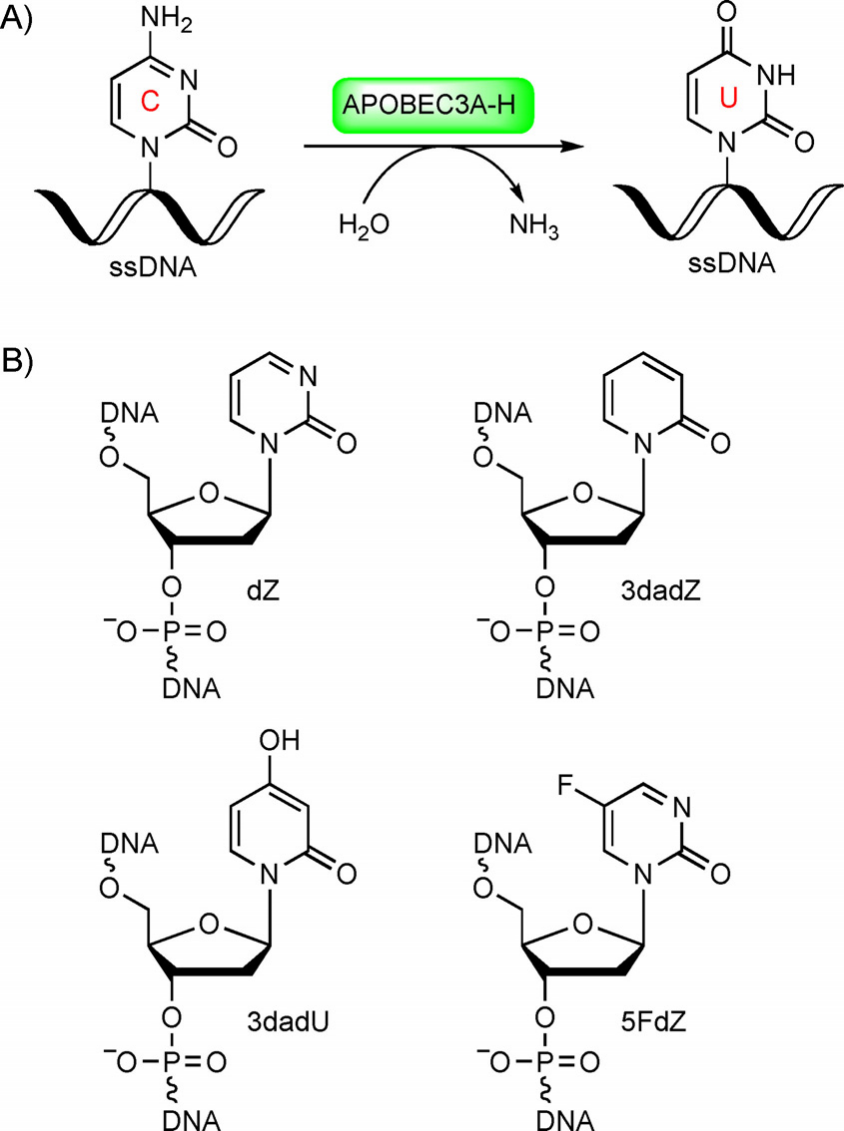
A) Deamination of dC in ssDNA through the action of APOBEC3 enzymes. B) Modified nucleotides as potential inhibitors of cytidine deamination.

APOBEC3 (A3) enzymes are important components of the innate immune system that protect against pathogens by catalysing the deamination of cytosine residues in the single‐stranded DNA (ssDNA) of the invading viral genome to form uracil residues (Scheme 1 A).^1^ These A3 enzymes therefore restrict the spread of pathogenic genetic information. Conversely, however, A3 enzymes, in particular A3B, can mutate genomic DNA, especially in cancer cells, thereby contributing to cancer genome evolution, acquired drug resistance and poor survival prognosis in cases of multiple cancers (including breast, bladder, cervix, lung, head and neck).^2^ A3B inhibition thus presents a promising new strategy to complement existing anticancer therapies,^3^ because A3B is a nonessential protein.^4^

A3 enzymes and cytidine deaminase (CDA) share a similar structural topology (despite very low sequence identity), together with, more importantly, structurally homologous zinc‐containing active sites. These active sites each include a crucial Glu residue that functions as a general acid/base in the hydrolysis of cytosine.^5^ Consequently, A3s and CDA share a similar mechanism of cytosine deamination. However, CDA accepts only individual nucleosides as substrates,^6^ whereas A3 enzymes have minimum ssDNA substrates of two or three nucleotides flanking the cytosine.^7^

To date, no selective small‐molecule inhibitors of A3A or A3B have been reported. We recently developed the first rationally designed competitive inhibitor of A3 enzymes by incorporating a known inhibitor of CDA—2′‐deoxyzebularine (dZ, Scheme 1 B)—into ssDNA oligonucleotides.^8^ We demonstrated that dZ does not inhibit A3 enzymes when present as the free nucleoside, but becomes a low‐micromolar inhibitor if, and only if, it is incorporated into ssDNA. This key observation represents support for a mechanism in which the ssDNA delivers the dZ into the active site for inhibition.

We propose that the inhibitory potential of ssDNAs can be further improved through the incorporation of potent inhibitors of CDA (also an enzyme of pharmaceutical interest)^9^ into ssDNA. Here we have considered several cytidine derivatives known to inhibit CDA and incorporated them into ssDNA as possible inhibitors of A3 enzymes (Scheme 1 B). 3‐Deazauridine (the ribose analogue of 3dadU) has been reported as a weak inhibitor of human liver CDA (*K*_i_=100 μm).^10^ 5‐Fluorozebularine has been shown to be a more potent inhibitor of mouse kidney CDA than zebularine (*K*_i_=0.3 μm versus 2.3 μm, respectively).^11^ However, RNA molecules are not preferred substrates of A3 enzymes.^12^

Herein, we report the first syntheses of the 2′‐deoxy forms of 3‐deazauridine and 5‐fluorozebularine (3dadU and 5FdZ, respectively). We also report the incorporation of these nucleosides into ssDNA and their evaluation as A3 inhibitors with the aid of our previously described NMR‐based7a, 8, 13 and fluorescence‐based^14^ enzymatic assays. 3‐Deaza‐2′‐deoxyzebularine (3dadZ, Scheme 1 B) has a CH motif instead of the N3 atom in comparison with dZ and so can be used to evaluate the importance of protonation of the N3 atom in dZ in its inhibitory mechanism. Our results indicate subtleties in inhibition of cytosine deamination catalysed by different A3 enzymes, and support our general strategy of using known inhibitors of CDA to guide the design of ssDNAs as inhibitors of A3 enzymes.

## Results and Discussion

### Synthesis of modified nucleosides, their DMT‐protected phosphoramidites and corresponding oligos

The synthesis of modified nucleosides started from heterocycles **1 a**–**c** and Hoffer's chlorosugar^15^ (Table 1). For the synthesis of 3dadZ (compounds **2**–**5 a**) and its incorporation into DNA, we followed previously described procedures^16^ with some modifications as described in the Supporting Information.

**Table 1:** Synthesis of modified nucleosides.

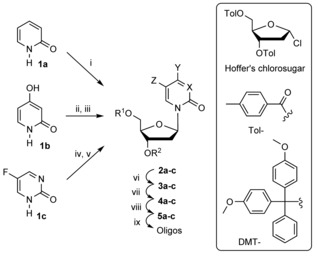						
	X	Y	Z	R^1^	R^2^	Yield
**2 a**	CH	H	H	Tol	Tol	11 %
**2 b**	CH	OH	H	Tol	Tol	50 %
**2 c**	N	H	F	Tol	Tol	45 %
						
**3 a**	CH	H	H	H	H	n.d.
**3 b**	CH	OH	H	H	H	quant.
**3 c**	N	H	F	H	H	n.d.
						
**4 a**	CH	H	H	DMT	H	58 % from **2 a**
**4 b**	CH	OBz	H	DMT	H	81 % from **2 b**
**4 c**	N	H	F	DMT	H	60 % from **2 c**
						
**5 a**	CH	H	H	DMT	P(N*i*Pr)_2_OCH_2_CH_2_CN	93 %
**5 b**	CH	OBz	H	DMT	P(N*i*Pr)_2_OCH_2_CH_2_CN	84 %
**5 c**	N	H	F	DMT	P(N*i*Pr)_2_OCH_2_CH_2_CN	89 %

i) Hoffer's chlorosugar, KOH, CH_3_CN, 15 min; ii) hexamethyldisilazane (HMDS), (NH_4_)_2_SO_4_ (cat), reflux 1 h; iii) Hoffer's chlorosugar, CHCl_3,_ distill., 15 min; iv) HMDS, (NH_4_)_2_SO_4_ (cat), reflux 1 h; v) Hoffer's chlorosugar, SnCl_4_, 1,2‐dichloroethane, −35 °C; vi) for **3 a** and **3 b**: 28 % aq. ammonia, MeOH, 48 h; for **3 c**: sat. ammonia in MeOH, 48 h; vii) for **4 a** and **4 c**: DMT‐Cl, pyridine, 0 °C→RT, overnight; for **4 b**: DMT‐Cl, pyridine, 0 °C→RT, overnight then Bz_2_O, pyridine, 0 °C→RT, overnight; viii) *N*,*N*‐diisopropylamino‐2‐cyanoethoxychlorophosphine, Et_3_N, CH_2_Cl_2_; ix) DNA synthesis and purification.

The pure β‐anomer of 3dadU (compound **2 b**) was obtained by use of a silyl modification of the classical Hilbert–Johnson reaction,^17^ by treating silylated 2,4‐dihydroxypyridine with Hoffer's chlorosugar in boiling CHCl_3_ (Supporting Information). Double recrystallisation from EtOH provided **2 b** in 50 % yield. Cleavage of the toluoyl protecting groups was accomplished in MeOH/NH_4_OH to provide nucleoside **3 b**,^18^ and this was then converted into **4 b** by selective installation of the 4,4′‐dimethoxytrityl (DMT) group on the 5′‐end of the nucleoside followed by benzoyl protection of the 4‐hydroxy group of the nucleobase (81 % yield over three steps from **2 b**). Phosphitylation of **4 b** was performed under standard conditions with *N*,*N*‐diisopropylamino‐2‐cyanoethoxychlorophosphine and Et_3_N in CH_2_Cl_2_, in 84 % yield after silica gel column chromatography.

The synthesis of 5FdZ as a free nucleoside has been performed in the past through enzymatic conversion of dC in the presence of heterocycle **1 c** with *trans*‐*N*‐deoxyribosylase from *Lactobacillus acidophilus*.^19^ Later, 5FdZ was synthesised from 5‐fluoro‐2′‐deoxyuridine in six steps.^20^ We found both protocols to be unsatisfactory in terms of potential scalability, complex procedures and overall yield. As with the syntheses of dZ^8^ and 3dadU, we first used a Lewis‐acid‐free variant of the silyl‐Hilbert–Johnson reaction for the preparation of 5FdZ from silylated heterocycle **1 c** and Hoffer's chlorosugar; this procedure failed.

Instead, it was necessary to use freshly distilled SnCl_4_ and low temperatures (−35 °C)^21^ to obtain 3′,5′‐bis‐*O*‐toluoyl‐protected 5FdZ (**2 c**) in a good yield, although this product was contaminated with the α‐anomer (β/α 9:1). Use of a slow stepwise gradient of acetone (0→20 %) in CH_2_Cl_2_ allowed isolation of pure β‐anomer **2 c** in 45 % yield. Deprotection was performed with saturated NH_3_ in MeOH, providing 5FdZ nucleoside **3 c**, an analytical sample of which was obtained after preparative TLC on silica gel.

**Figure 1:**
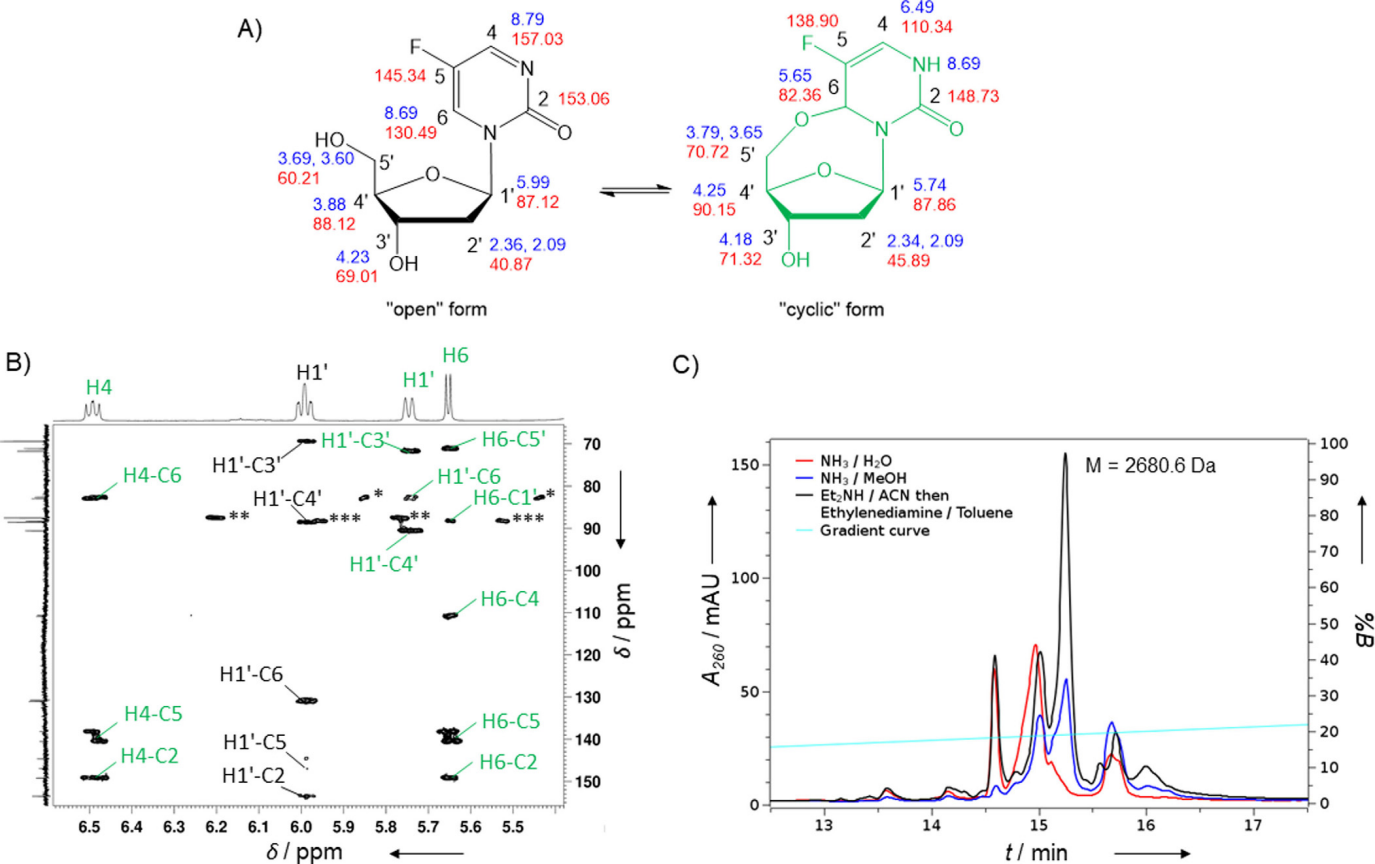
A) NMR assignment of “open” (structure in black) and “cyclised” (structure in green) forms of 5FdZ. Chemical shifts (*δ* in ppm) are shown for ^1^H in blue and for ^13^C in red. B) ^1^H,^13^C HMBC spectrum recorded in [D_6_]DMSO at RT, showing two‐ to four‐bond correlations and coexistence of “open” (black labels) and “cyclised” (green labels) forms of 5FdZ. *, **, and ***: single‐bond correlations of H6−C6 (“cyclised” form), H1′−C1′ (“open” form) and H1′−C1′ (“cyclised” form), respectively. C) RP‐HPLC profiles of 5FdZ‐oligo cleaved from the support and deprotected under different conditions. The major peak isolated after treatment with Et_2_NH/CH_3_CN followed by ethylenediamine/toluene gave the expected mass (ESI‐MS) of 2680.6 Da (calcd. for [*M*]: 2680.5 Da).

NMR analysis revealed that this compound exists in two forms: as an “open” nucleoside and as a “cyclic” nucleoside formed after intramolecular addition of the 5′‐OH group to the double bond of the nucleobase (Figure 1 A). Similar transformations have been described for several pyrimidines.^22^ Using 2D NMR techniques, we assigned signals in ^1^H and ^13^C NMR spectra for individual forms as reported in the Supporting Information and shown in Figure 1 A. The appearance of an NH signal at 8.69 ppm in the ^1^H NMR spectrum and significant shifts in the H6 and C6 signals in the ^1^H and ^13^C NMR spectra as a result of a change in hybridisation at C6 suggest the formation of a “cyclic” nucleoside. The ^1^H,^13^C HMBC spectrum, which shows three‐bond correlations, was particularly helpful during the assignment (Figure 1 B). The H6−C5′ crosspeak, seen in the right‐hand upper corner of Figure 1 B, confirms the existence of a three‐bond linkage between H6 of the nucleobase and C5′ of the sugar in the “cyclic” nucleoside. At the same time, the H6 proton cross‐talks with the other carbon atoms of the nucleobase (C2, C4, C5) and with the C1′ carbon atom of the sugar moiety; this is possible only for an O5′−C6 “cyclic” nucleoside.

We observed that the ratio between “open” and “cyclic” forms changes in different solvents. In D_2_O, the “open” form predominantly exists, whereas in [D_6_]DMSO, CD_3_CN and [D_8_]THF both forms are present. This indicates that “open” and “cyclic” forms of the 5FdZ nucleoside are in dynamic equilibrium, which complicates purification but allows the transformation of an equilibrium mixture of nucleosides into the single 5′‐DMT‐modified product **4 c**. Consequently, “open” and “cyclic” forms of nucleoside **3 c**, without any purification after the removal of toluoyl groups from **2 c**, were treated with DMT‐Cl in pyridine; compound **4 c** was obtained in 60 % yield over two steps from **2 c**. Finally, phosphitylation of **4 c** gave phosphoramidite **5 c** in 89 % yield.

We incorporated the modified nucleosides at the location of dC in the preferred A3 substrate motifs. A3B and A3A prefer the TCA motif (oligo1, Table 2) whereas A3G preferentially catalyses deamination of the CCCA motif (oligo2, in which the underlined C is deaminated first). The synthesis of DNA oligos was performed with an automated DNA synthesiser and use of an increased coupling time for phosphoramidites **5 a**–**c**, from 1.5 min for standard phosphoramidites to 5 min.

**Table 2:** Oligonucleotides used in this study.

Name	Sequence 5′→3′
**Oligos used in NMR‐based activity assay**	
oligo1	ATTT‐C‐ATTT
oligo2	ATTCC‐C‐AATT
dZ‐oligo^[a]^	ATTT‐dZ‐ATTT
3dadZ‐oligo	ATTT‐3dadZ‐ATTT
3dadU‐oligo	ATTT‐3dadU‐ATTT
5FdZ‐oligo	ATTT‐5FdZ‐ATTT
CC5FdZ‐oligo	ATTCC‐5FdZ‐AATT
**Oligos used in fluorescence‐based activity assay**	
T4‐dZ‐oligo^[a]^	TTTT‐dZ‐AT
T4‐5FdZ‐oligo	TTTT‐5FdZ‐AT

[a] Prepared as in ref. 8.

In the cases of oligos containing 3dadU and 3dadZ, cleavage from the solid support and deprotection of phosphates and nucleobases was accomplished in concentrated aqueous NH_4_OH. Unfortunately, the same procedure led to degradation of 5FdZ‐containing oligos, as is evident from the reversed‐phase HPLC profile in Figure 1 C (red line). Attempted deprotection with saturated NH_3_ in MeOH was also unsuccessful (blue line, Figure 1 C). We found that on‐column deprotection of 5FdZ‐oligo in organic solvents^23^ led to the least amounts of by‐products (black profile, Figure 1 C). Here, 5FdZ‐oligo on the CPG support was treated with 10 % Et_2_NH in acetonitrile for 5 min, followed by incubation of the support in an ethylenediamine/toluene mixture for 2 h at room temperature, allowing subsequent release of the deprotected oligo in H_2_O. All oligos were purified by reversed‐phase HPLC. Their compositions were confirmed by ESI‐MS (see the Supporting Information).

### Evaluation of oligos as inhibitors of A3 enzymes by using an NMR‐based activity assay

To assess the inhibition of A3 enzymes directly, we used a previously described NMR‐based activity assay in which the DNA substrate deamination is monitored by ^1^H NMR spectroscopy in the presence of enzyme with and without inhibitors.7a, 8, 13 The NMR‐based inhibition assay is a direct assay using just A3 enzymes; it does not require a secondary enzyme, such as uracil‐DNA glycosylase (UDG), as used in many indirect assays. By introducing different inhibitors of cytidine deamination into the A3 recognition motif preferred by the particular A3 enzyme, we expected that the trend in inhibition for all A3 enzymes would roughly parallel the trend observed for CDA inhibition, because the active site and therefore the deamination mechanism are conserved. We evaluated the inhibitory activity of our modified DNAs by using active A3 enzymes that displayed reliable expression and stability over time and had also been characterised previously in the NMR‐based activity assays in our laboratory. This allows reliable determination of the inhibitory potential of modified oligos through comparison of *K*_m_ values of the substrates with *K*_i_ values of inhibitors determined under identical conditions (enzyme and substrate concentrations, buffer and ionic strength). The enzymes chosen—A3B_CTD_‐QM‐ΔL3‐AL1swap (hereafter simplified to A3B_CTD_‐AL1) and GST‐fused A3G_CTD_—were recombinantly expressed and purified from *Escherichia coli*. To compare the inhibitory effect of oligonucleotides between A3G_CTD_ and full‐length A3G (flA3G) we used flA3G that was purified from human cells grown planktonically^24^ (see description of these enzymes and their purification in the Supporting Information).

**Figure 2:**
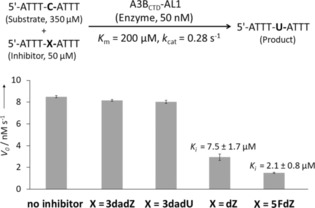
Inhibition of A3B_CTD_‐QM‐ΔL3‐AL1swap‐catalysed deamination of the substrate (5′‐ATTT‐**C‐**ATTT) by oligos containing modified nucleosides. The bold **C** denotes the target 2′‐deoxycytidine residue deaminated through the action of the enzyme. Determination of *K*_i_ for 5FdZ‐oligo can be found in the Supporting Information. The *K*_i_ (mean and standard deviation) for the dZ‐oligo was reported previously^8^ and is provided for comparison; all experiments were repeated multiple times in the same laboratory and with the same time interval. Mean values are plotted. The error bars report standard deviations.

Oligos containing 3dadZ and 3dadU in place of the target dC component in the preferred TCA‐recognition motif for A3B_CTD_‐AL1 had no effect on the initial speed of deamination catalysed by A3B_CTD_‐AL1 (Figure 2). These oligos fail to inhibit A3 enzymes under experimental conditions. These data are in line with previous findings that 3dadU, as an individual ribose‐based single nucleoside, is a very weak inhibitor of human liver CDA (*K_i_*=100 μm).^10^ Although higher concentrations of 3dadU‐oligo might result in inhibition of A3B_CTD_‐AL1, the use of such concentrations would provide a weaker basis than our current strategy for the development of modified 3dadU‐oligos as inhibitors.

**Scheme 2:**
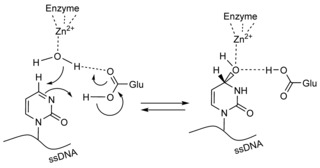
Proposed conversion of dZ into its hydrate and formation of a transition‐state analogue of cytosine deamination.

On the other hand, inhibition of A3B_CTD_‐AL1 by 5FdZ‐oligo was more powerful than that by dZ‐oligo under identical conditions. Previously, we had confirmed that dZ‐oligo is a competitive inhibitor of this enzyme.^8^ By monitoring the reaction in the presence of inhibitor at various concentrations, we obtained the inhibition constant (*K*_i_) for 5FdZ‐oligo [(2.1±0.8) μm, Supporting Information]; this was 3.5 times lower than the *K*_i_ of dZ‐oligo [(7.5±1.7) μm]. The overall inhibition effect was improved from 30‐fold (dZ) to nearly 100‐fold (5FdZ) if the apparent inhibition constants (*K*_i_) of dZ‐ and 5FdZ‐containing oligos are compared with the *K*_m_ of the ssDNA substrate 5′‐ATTT‐C‐ATTT (*K*_m_=200 μm). This means that 5FdZ‐containing oligos can potentially be used in cells in the low‐micromolar range to inhibit A3A and A3B. Thanks to the presence of the electron‐withdrawing F, the heterocycle component in 5FdZ is more activated towards the nucleophilic addition of H_2_O than its counterpart in the case of dZ (Scheme 2), as is evident from the existence of 5FdZ in equilibrium between “open” and “closed” forms (Figure 1 A). This probably explains why 5FdZ, once embedded in the ssDNA, is a better inhibitor of A3B_CTD_‐AL1 than dZ. Formation of reversible covalent adducts with the enzyme is also possible. Similar adducts between zebularine and DNA methyltransferases have been described.^25^

Our observations parallel those reported earlier for CDA: that is, that 5‐fluorozebularine is a better inhibitor than zebularine and 3‐deazauridine (*K*_i_=0.3, 2.3^11^ and 100 μm,^10^ respectively). These results indicate that the structure of the nucleoside used in place of dC in the preferred ssDNA substrate determines the inhibitory potential of the oligos and that the trend of A3B_CTD_‐AL1 inhibition correlates with *K*_i_ values reported earlier for individual nucleosides against CDA. This result also opens the possibility of further improvement of inhibition by introducing other inhibitors of cytidine deamination into ssDNA sequences.

The fact that 3dadZ does not inhibit A3B_CTD_‐AL1 highlights the importance of protonation of N3 in dZ by the conserved glutamic acid residue present in the active site of A3s (and CDA). This protonation makes C4 in dZ more electrophilic and more susceptible to nucleophilic attack by OH^−^/H_2_O coordinated to the Zn^2+^, which converts dZ into a tetrahedral transition‐state analogue of cytidine deamination (Scheme 2).^26^ This mechanism is inoperative in the case of 3dadZ, because the C=C double bond of 3dadZ is inactive towards water addition. Moreover, the nucleobase of 3dadU is planar and does not mimic the tetrahedral geometry of C4 in the transition state of cytidine deamination.

**Figure 3:**
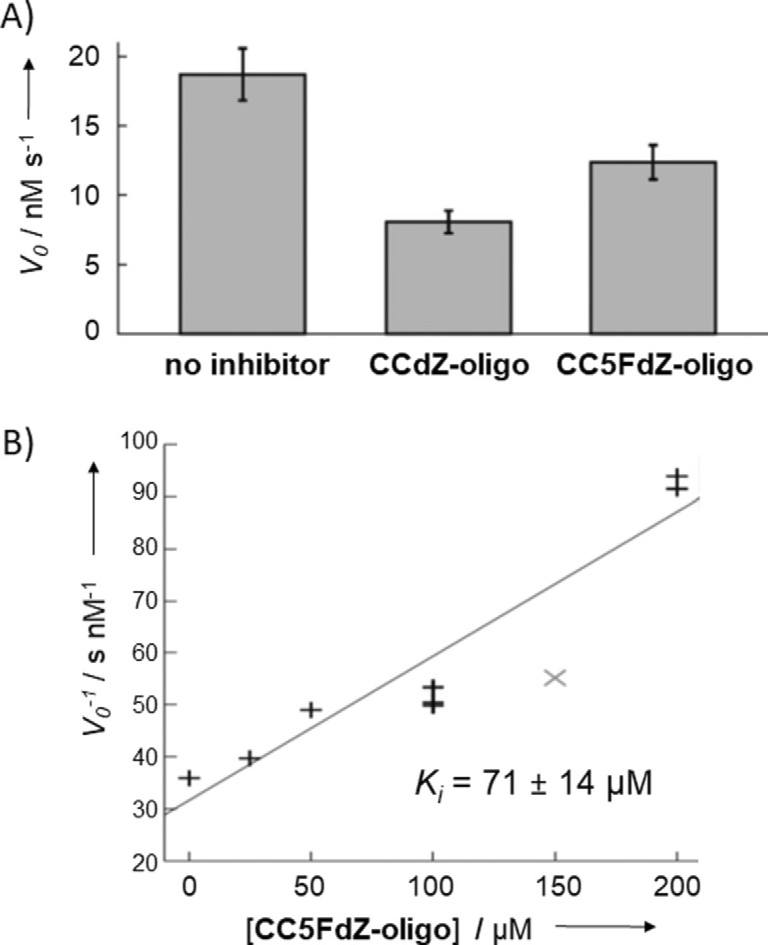
A) Speed of deamination of the substrate (5′‐ATTCCCAATT, 500 μm) catalysed by full‐length A3G in the absence of inhibitor and in the presence of CCdZ‐ and CCFdZ‐oligos (50 μm). Conditions: 100 mm NaCl, 50 mm sodium phosphate buffer, pH 6.0, 10 % D_2_O, 1 mm citrate supplemented with 50 μm 3‐trimethylsilylpropanesulfonic acid (TMSPS) as standard. Error bars represent standard deviations. B) Dixon plot of inverse speed of deamination against inhibitor concentration for A3G_CTD_‐catalysed deamination of 5′‐ATTCCCAATT (320 μm, underlined C is the one deaminated) in the presence of CC5FdZ‐oligo. The grey point was ignored by fitting as an outlier according to the Q‐test (with 95 % confidence).

Next, having two active A3G enzymes—the C‐terminal domain (A3G_CTD_) with the wild‐type sequence and full‐length A3G (flA3G)—we decided to test whether inhibition of A3G_CTD_ is a good model for investigation of inhibition of two‐domain enzymes such as flA3G. Our studies were performed with two oligos: an A3G‐preferred CC5FdZ‐oligo in which the dC residue that is first deaminated by A3G was changed to 5FdZ, and the previously reported inhibitor CCdZ‐oligo.^8^ Our data show that inhibition of A3G deaminase activity by targeting only the catalytically active C‐terminal domain, A3G_CTD_, accurately translates to the overall inhibition of flA3G (Figure 3 A). This is consistent with the fact that the N‐terminal domain of A3G completely lacks deaminase activity.^27^ Accordingly, the specificity of ssDNA binding to the full‐length A3G, and by implication A3B, lies in the C‐terminal domains, and the catalytically inactive N‐terminal domains enhance ssDNA deamination efficiency at the C‐terminal domain and regulate processivity of enzymes.27a, 28

Interestingly, the CC5FdZ‐oligo [*K*_i_=(71±14) μm, Figure 3 B] did not cause greater inhibition of A3G_CTD_ in comparison with CCdZ‐oligo [*K*_i_=(53±10) μm],^8^ in contrast to the trend observed above for A3B_CTD_‐AL1. Nonetheless, the fact that both CCdZ‐oligo and CC5FdZ‐oligo are inhibitors supports our strategy of targeting the catalytically active C‐terminal domains of A3 enzymes with our DNA‐based inhibitors as a means to inhibit full‐length enzymes.

### Evaluation of oligos as inhibitors of A3A by use of a fluorescence‐based activity assay

**Figure 4:**
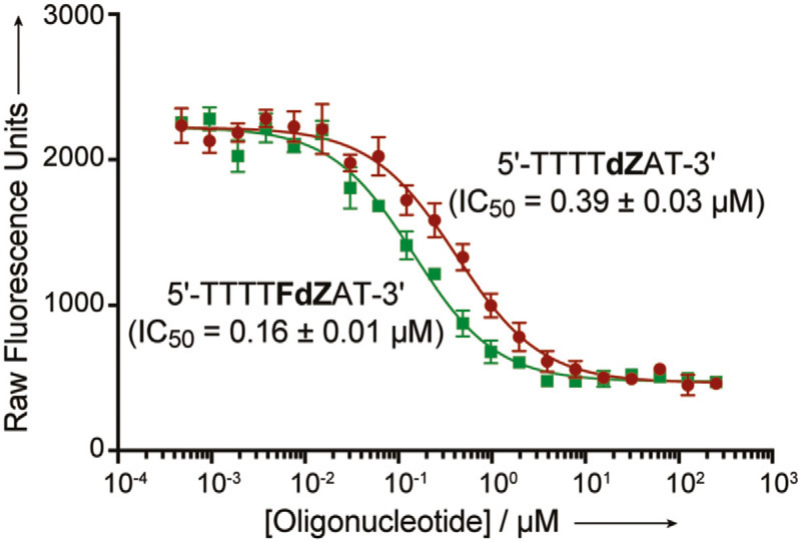
A) Inhibition of deamination of a fluorescently labelled oligonucleotide reporter, catalysed by human A3A, by dZ‐ and 5FdZ‐containing oligonucleotide competitors. Representative graphic data are shown. Individual replicates and the sequence of the fluorescent oligonucleotide reporter are provided in the Supporting Information. IC_50_ values are means± SEMs for four biological replicates.

To investigate how a 5FdZ‐containing oligo would inhibit another wild‐type A3 expressed in human cells, we purified A3A from HEK293T and used it to perform our previously published fluorescence‐based activity assay.^14^ The deamination of a fluorescently labelled substrate oligonucleotide in the presence of dZ‐ and 5FdZ‐containing oligo competitors was monitored (Figure 4). The assay had been developed previously to evaluate small‐molecule inhibitors of A3A, A3B and A3G in identical settings. In this work, we used an 18‐mer oligo with 5′‐…TATCCCA…‐3′ in the middle of the sequence as the enzyme substrate (Supporting Information). The CCC motif is a preferred sequence for deamination through the action of A3G^28^, ^29^ but this sequence is also readily deaminated in the presence of A3A and A3B;^30^ therefore, the oligo is a pan‐A3 substrate. The results clearly show that, in the case of A3A, the 5FdZ‐containing oligo is a more potent inhibitor, with an IC_50_ of 0.16±0.01 μm, compared to the equivalent dZ‐containing oligonucleotide sequence with an IC_50_ of 0.39±0.03 μm. These data are consistent with the A3B_CTD_‐AL1 data, because in X‐ray structures A3A and A3B_CTD_ share all the residues surrounding the target cytosine residue.^31^ Control assays with the dC‐ and dU‐containing oligos can be found in the Supporting Information.

### Plausible explanation of differences in inhibition of A3A/A3B_CTD_ and A3G by dZ‐ and 5FdZ‐containing oligos

The fact that the trend of inhibition by dZ‐ and 5FdZ‐containing oligos is varied for A3A/A3B_CTD_ and A3G should not be completely unexpected, because A3 family members differ strongly in their preferences for deamination of methylated cytosine residues in ssDNA.^32^ The selectivity of different A3 family members for nonmethylated versus 5‐methylated 2′‐deoxycytidine (5MedC) can be significantly changed by swapping loop 1 and loop 7 between the enzymes.5b, 33 This property suggests subtle control of the active site specificity for deamination of 5MedC, because the swapped amino acids are not in direct contact with the target cytosine moiety. Similar effects can be relevant to interaction between A3 enzymes and 5‐fluoro‐2′‐deoxycytidine (5FdC) or 5FdZ. Recently, we have also observed that 5‐methyl‐2′‐deoxyzebularine is a worse inhibitor than dZ in the context of an oligo designed to inhibit A3G_CTD_.^8^

Nevertheless, we have compared active sites of A3A, A3B and A3G to find possible differences in amino acids in proximity to the target cytosine residue. Such differences might explain the preferences of A3 enzymes towards various substrates and inhibitors.

Structural analysis of the A3B_CTD_‐AL1 complex with ssDNA^31^ and sequential alignment of A3A, A3B_CTD_ and A3G_CTD_ revealed that not only the zinc‐coordinating residues, but most of the residues in the active site close to the target cytosine moiety, are well conserved between these proteins. However, one residue in the substrate‐binding pocket differs: an isoleucine residue, Ile279/Ile96, in A3B_CTD_/A3A is a threonine residue (Thr283) in the corresponding position of the A3G_CTD_ sequence. The side chain of Ile279 is ≈4.4 Å distant from the NH_2_ group of the target cytosine moiety in the inactive, substrate‐binding E255A mutant of A3B_CTD_ (PDB ID: 5TD5). On the other hand, the Thr283 hydroxy group makes hydrogen bonds to a neighbouring Thr residue and to the main chain that forms part of the substrate/inhibitor binding pocket (PDB ID: 3V4J). This interaction might reduce the conformational flexibility needed to accommodate a substituent larger than hydrogen in the 5‐position of cytosine. We note that in the A3B_CTD_–AL1 structure the bound cytidine residue is tightly supported by Tyr313 (Tyr315 in A3G_CTD_), and that this in turn is buttressed by a conserved Trp residue (Trp285 in the case of A3G_CTD_) on the loop that, in the case of A3G_CTD_, is locked in place by hydrogen bonding to Thr283. Interestingly, mouse CDA (PDB ID: 2FR5) has Ile87 in a similar position in the three‐dimensional structure to that of Ile279 in A3B_CTD_ (Figure S3 in the Supporting Information). Thus, the Ile versus Thr substitution might play a role in the differences seen between 5FdZ and dZ inhibition of A3G_CTD_ and A3B_CTD_‐AL1. AID, mouse APOBEC1 and mouse APOBEC3 catalyse the deamination both of 5FdC and of 5MedC less efficiently than for dC.32b, 34 These results were explained in terms of steric effects, because F and Me are larger than H. As discussed above, the active sites around the target cytosine residue are very similar, with the exception of Ile/Thr, in the cases of A3A/A3B_CTD_ and of A3G_CTD_. The dynamics of the active sites might allow better accommodation of 5FdZ in A3A/A3B_CTD_ than in A3G_CTD_. In any event, the substrate and inhibitor binding and the deamination mechanism vary subtly between A3s and CDAs. Examples of highly homologous enzymes with significantly different transition states are well‐established.^35^

## Conclusion

The structures of modified nucleosides dZ and 5FdZ embedded in the otherwise identical DNA sequence determine the inhibitory effects on human A3A, A3B_CTD_, and A3G_CTD_, as well as on full‐length A3G. On the other hand, the 2′‐deoxyribosyl derivative of 3‐deazauridine, a previously described weak inhibitor of CDA, cannot inhibit A3 upon its incorporation into ssDNA under the conditions tested. Our results indicate that some correlation between CDA and A3 inhibition exists when CDA inhibitors replace the deamination‐susceptible cytidine moiety in the ssDNA sequence. Our results also highlight the importance of protonation of the N3 atom in dZ for its inhibitory behaviour. Noteworthy differences in inhibition profiles among different A3 enzymes observed here point to possibilities of obtaining highly specific A3 inhibitors, thereby supporting our approach to development of oligonucleotide‐based A3 inhibitors with the aid of chemically modified nucleosides, the structures of which can stall enzymatic cytosine deamination.^36^ Future work will continue to focus on the chemical optimisation of our ssDNA‐based A3 inhibitors and their evaluation in vitro and in vivo. Nucleotides flanking the target dZ and 5FdZ motifs can be further modified to improve inhibitory potential and to enhance the lifetimes of oligonucleotides in biological media.

## Conflict of interest

*D.A.H. is a co‐founder, shareholder and consultant of ApoGen Biotechnologies, Inc*.

## Supporting information

As a service to our authors and readers, this journal provides supporting information supplied by the authors. Such materials are peer reviewed and may be re‐organized for online delivery, but are not copy‐edited or typeset. Technical support issues arising from supporting information (other than missing files) should be addressed to the authors.

Supplementary
