# Supplementary material for: Differential Inhibition of APOBEC3 DNA‐Mutator Isozymes by Fluoro‐ and Non‐Fluoro‐Substituted 2′‐Deoxyzebularine Embedded in Single‐Stranded DNA
Source: Chembiochem. 2019 Dec 19;21(7):1028–35. doi: 10.1002/cbic.201900505 (PMC7142307; doi:10.1002/cbic.201900505)
Supplement: Supplementary file 1 — Supplementary [file CBIC-21-1028-s001.pdf]

CHEMBIOCHEM

Supporting Information

Differential Inhibition of APOBEC3 DNA-Mutator Isozymes by Fluoro- and Non-Fluoro-Substituted 2′-Deoxyzebularine Embedded in Single-Stranded DNA

Maksim V. Kvach,[a] Fareeda M. Barzak,[a] Stefan Harjes,[a] Henry A. M. Schares,[c] Harikrishnan M. Kurup,[a, b] Katherine F. Jones,[d] Lorraine Sutton,[f] John Donahue,[f] Richard T. D′Aquila,[g] Geoffrey B. Jameson,[a, b] Daniel A. Harki,[c, d] Kurt L. Krause,[b, e] Elena Harjes,*[a, b] and Vyacheslav V. Filichev*[a, b]

cbic_201900505_sm_miscellaneous_information.pdf

Contents

1. Synthesis of transition state analogues of cytidine deamination 2

1.1. General Methods 2

1.2. Synthetic procedures 2

2. Synthesis of modified oligodeoxynucleotides (oligos) 13

3. Protein expression and purification 20

4. Evaluation of inhibitors in NMR-based assay 23

5. Evaluation of inhibitors in fluorescence-based APOBEC3A ssDNA deamination assay and UDG inhibition control experiments 25

6. References: 27

1. Synthesis of transition state analogues of cytidine deamination

1.1. General Methods

Reagents from commercial suppliers were used without further purification. Pyridine, Et_3N and CH_2Cl_2 were freshly distilled from CaH_2. Hoffer's chlorosugar was prepared as described.[1] ^{1}H (400.1, 500.1 or 700.1 MHz), ^{13}C (100.6, 125.8 or 176.1 MHz), ^{19}F (376.5 MHz) and ^{31}P (202.5 MHz) NMR spectra were obtained with Bruker Avance 400, Avance 500 or Avance 700 MHz spectrometers and referenced to residual solvent signals ($CDCl_3$: 7.26 ppm for ^{1}H and 77.16 ppm for ^{13}C, d_6-DMSO: 2.50 ppm for ^{1}H and 39.52 ppm for ^{13}C according to reference[2], neat $CFCl_3$ external standard (0.0 ppm for ^{19}F) or 85% aq. H_3PO_4 external standard (0.0 ppm for ^{31}P)). ^{1}H NMR coupling constants are reported in Hertz (Hz) and refer to apparent multiplicities. The assignments of signals were done using 2D homonuclear ^{1}H-^{1}H COSY, NOESY and heteronuclear ^{1}H–^{13}C HMQC or HSQC, and HMBC spectra. NMR spectra were processed in Spinworks ver. 4.1 (developed by Dr. Kirk Marat, Department of Chemistry, University of Manitoba) or TopSpin ver. 2.1 (Bruker). Mass spectra were recorded on a Thermo Scientific MSQ Plus mass spectrometer. High-resolution electrospray mass spectra were recorded on a Thermo Scientific Q Exactive Focus Hybrid Quadrupole-Orbitrap mass spectrometer. Raw-files were converted to open mzML format using ProteoWizard tools[3] and processed in mMass software[4]. Analytical thin-layer chromatography was performed on Kieselgel 60 F_{254} precoated aluminum plates (Merck). Silica gel column chromatography was performed using silica gel 60 (40–63 µm).

1.2. Synthetic procedures

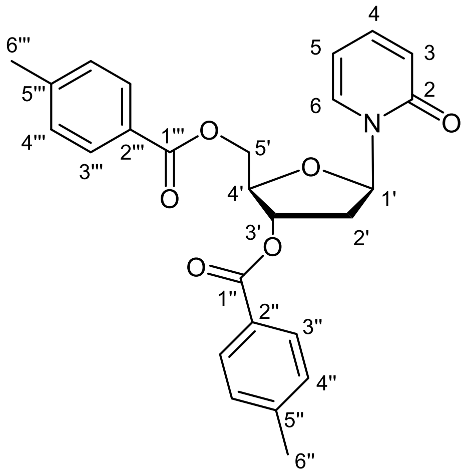

3',5'-Di-*O*-(p-toluoyl)-2'-deoxy-3-deazazebularine (2a) was prepared similarly to the procedure described earlier.[5] 2-Hydroxypyridine (1.9 g, 20 mmol) was dissolved in acetonitrile and KOH (1.9 g, 34 mmol) was added in one portion. After stirring for 5 min, Hoffer's chlorosugar (7.78 g, 20 mmol) was added. Reaction mixture was stirred for 15 min, filtered and precipitate washed with acetonitrile. Combined acetonitrile fractions were evaporated *in vacuo*. The residue was purified on silica gel column chromatography (100 g silica gel) eluting with a step gradient of acetone (0→30%) in CH_2Cl_2 to yield a fraction (1.66 g, 18%) containing both α- and β-anomers and pure α-anomer (2.88 g, 32%). Pure β-anomer of **2a** (0.95 g, 11%) was isolated after second chromatography separation. R_f 0.39 (α-anomer), 0.51 (β-anomer) (EtOAc/n-hexane, 1/1).

^{1}H NMR (500 MHz, d_6-DMSO) δ 7.92 (m, 2H, H-3''), 7.84 (m, 2H, H-3'''), 7.75 (ddd, 1H, $J_{5,6}$ = 7.1 Hz, $^{4}J_{4,6}$ = 2.1 Hz, $^{5}J_{3,6}$ = 0.7 Hz, H-6), 7.43 (ddd, 1H, $J_{3,4}$ = 9.2 Hz, $J_{4,5}$ = 6.5 Hz, $^{4}J_{4,6}$ = 2.1 Hz, H-4), 7.35 (m, 2H, H-4''), 7.30 (m, 2H, H-4'''), 6.49 (dd, 1H, $J_{1',2'\beta}$ = 7.9 Hz, $J_{1',2'\alpha}$ = 6.0 Hz, H-1'), 6.41 (ddd, 1H, $J_{3,4}$ = 9.2 Hz, $^{4}J_{3,5}$ = 1.4 Hz, $^{5}J_{3,6}$ = 0.7 Hz, H-3), 6.22 (dt, 1H, $J_{4,5}$ = $J_{5,6}$ = 6.8 Hz, $^{4}J_{3,5}$ = 1.4 Hz, H-5), 5.60 (dt, 1H, $J_{2'\beta,3'}$ = $J_{3',4'}$ = 6.6 Hz, $J_{2'\alpha,3'}$ = 2.5 Hz, H-3'), 4.65-4.61 (m, 2H, H-5'), 4.61-4.58 (m, 1H, H-4'), 2.71 (ddd, 1H, $J_{2'\alpha,2'\beta}$ = 14.4 Hz, $J_{1',2'\alpha}$ = 6.0 Hz, $J_{2'\alpha,3'}$ = 2.5 Hz, H-2'α), 2.41 (ddd, 1H, $J_{2'\alpha,2'\beta}$ = 14.4 Hz, $J_{1',2'\beta}$ = 7.9 Hz, $J_{2'\beta,3'}$ = 6.6 Hz, H-2'β), 2.39 (s, 3H, H-6''), 2.36 (s, 3H, H-6''').

^{13}C{^{1}H} NMR (125.7 MHz, d_6-DMSO) δ 165.5 (C1'''), 165.3 (C1''), 161.0 (C2), 144.1 (C5''), 144.0 (C5'''), 140.2 (C4), 133.0 (C6), 129.5 (2C, C3''), 129.369 and 129.362 (4C, C4'',4'''), 129.3 (2C, C3'''), 126.54 and 126.48 (2C, C2'',2'''), 119.8 (C3), 105.8 (C5), 85.2 (C1'), 82.0 (C4'), 74.9 (C3'), 64.2 (C5'), 37.8 (C2'), 21.22 and 21.18 (2C, C6'',6''').

ESI-MS: Calcd. for $[M+H]^+$: 448.1760, Found: 448.1689

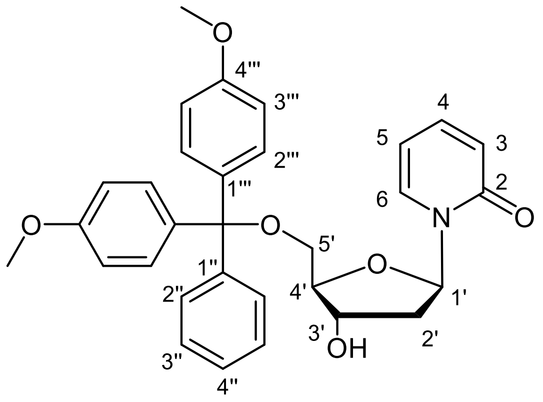

5'-*O*-(4,4'-Dimethoxytrityl)-2'-deoxy-3-deazazebularine (4a) was prepared similarly to the procedures described earlier.[6, 7] 3',5'-Di-*O*-(p-toluoyl)-2'-deoxy-3-deazazebularine **2a** (0.90 g, 2.0 mmol) was dissolved in MeOH (75 mL) and aq. ammonia (28%, 7.5 mL) was added in one portion. Reaction mixture was stirred at room temperature for 48 h, rotary evaporated *in vacuo*, co-evaporated with abs. pyridine (2 × 10 mL) to afford deprotected nucleoside **3a**. This was dissolved in abs. pyridine (20 mL), cooled to 0°C and 4,4'-dimethoxytritylchloride (0.75 g, 2.2 mmol) was added. Reaction was stirred overnight on a melting ice bath, evaporated *in vacuo*, residue dissolved in CH_2Cl_2 (100 mL), washed with H_2O (100 mL) and satd. $NaHCO_3$ (100 mL), filtered through Na_2SO_4 and rotary evaporated *in vacuo*. Flash chromatography on silica eluting with a step gradient of 2-propanol (0→4%) in a mixture of CH_2Cl_2/Et_3N 99:1 (v/v) afforded **4a** (0.60 g, 58% on **2a**) as a yellowish foam after rotary evaporation of solvent. R_f 0.42 (2-propanol/Et_3N/CH_2Cl_2, 5/1/94).

^{1}H NMR (500 MHz, d_6-DMSO) δ 7.78 (dd, 1H, $J_{5,6}$ = 7.1 Hz, $^{4}J_{4,6}$ = 2.0 Hz, H-6), 7.42-7.37 (m, 3H, H-4,2''), 7.34-7.29 (m, 2H, H-3''), 7.274, 7.270 (2d, 4H, $J_{2''',3'''}$ = 8.9 Hz, H-2'''), 7.25-7.21 (m, 1H, H-4''), 6.90 (d, 4H, $J_{2''',3'''}$ = 8.9 Hz, H-3'''), 6.38-6.34 (m, 2H, H-3,1'), 6.07 (dt, 1H, J = 6.8 Hz, $^{4}J_{3,5}$ = 1.3 Hz, H-5), 5.38 (d, 1H, $J_{3',OH}$ = 4.6 Hz, H-O*H*), 4.31 (m, 1H, H-3'), 3.98 (q, 1H, J = 4.0 Hz, H-4'), 3.734, 3.732 (2s, 6H, H-C*H*$_3$), 3.27 (d, 2H, $J_{4',5'}$ = 4.0 Hz, H-5'), 2.34 (ddd, 1H, $J_{2'\alpha,2'\beta}$ = 13.2 Hz, J = 6.4 Hz, J = 4.6 Hz, H-2'α), 2.04 (dt, 1H, $J_{2'\alpha,2'\beta}$ = 13.2 Hz, J = 6.4 Hz, H-2'β).

^{13}C{^{1}H} NMR (125.7 MHz, d_6-DMSO) δ 161.0 (C2), 158.2 (2C, C4'''), 144.7 (C1''), 139.9 (C4), 135.5 and 135.3 (2C, C1'''), 133.1 (C6), 129.80 and 129.79 (4C, C2'''), 127.9 (2C, C3''), 127.7 (2C, C2''),

126.8 (C4''), 119.5 (C3), 113.28 and 113.27 (4C, C3'''), 105.4 (C5), 85.8 (*C*Ar$_3$), 85.7 (C4'), 84.5 (C1'), 69.9 (C3'), 63.2 (C5'), 55.1 (2C, *C*H$_3$), 41.1 (C2').

ESI-MS: Calcd. for $[M+Na]^+$: 536.2049, Found: 536.2061

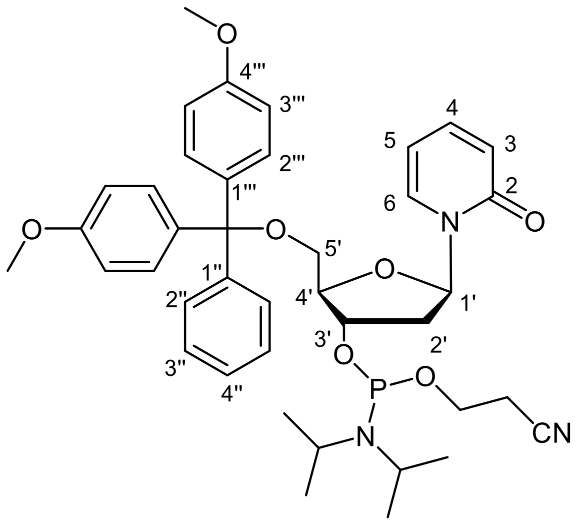

5'-*O*-(4,4'-Dimethoxytrityl)-3'-*O*-(*N*,*N*-diisopropylamino-2-cyanoethoxyphosphanyl)-2'-deoxy-3-deazazebularine (5a) was prepared similarly to the procedures described earlier.[6, 7] 5'-*O*-DMT-2'-deoxy-3-deazazebularine (**4a**, 0.56 g, 1.0 mmol) was co-evaporated with abs. CH_2Cl_2 (10 mL), dissolved in abs. CH_2Cl_2 (10 mL) and Et_3N (0.28 mL, 2.0 mmol) followed by *N*,*N*-di*iso*propylamino-2-cyanoethoxychlorophosphine (0.39 g, 1.6 mmol) were added under argon. After stirring for 15 min reaction was complete by TLC analysis (TLC in CH_2Cl_2/acetone/Et_3N, 90:5:5). Reaction mixture was diluted with CH_2Cl_2 (25 mL), washed with satd. $NaHCO_3$ (3 × 25 mL), filtered through Na_2SO_4 and rotary evaporated *in vacuo*. The residue was purified by flash chromatography on silica (packed in CH_2Cl_2/Et_3N, 90:10 and washed with CH_2Cl_2) eluting with a step gradient of AcOEt (0→1%) in CH_2Cl_2. Fractions with the product were collected, rotary evaporated *in vacuo*, co-evaporated with abs. CH_2Cl_2 and freeze-dried *in vacuo* from benzene to yield **5a** as a mixture of two diastereomers (0.73 g, 93%).

^{1}H NMR (500 MHz, d_6-DMSO) δ 7.83-7.76 (m, 1H, H-6); 7.43-7.36 (m, 3H, H-4,2''); 7.34-7.18 (m, 7H, H-3'',4'',2'''); 6.92-6.85 (m, 4H, H-3'''); 6.41-6.33 (m, 2H, H-3,1'); 6.12-6.06 (m, 1H, H-5); 4.59-4.50 (m, 1H, H-3'); 4.16-4.06 (m, 1H, H-4'); 3.735, 3.734, 3.730, 3.728 (4s, 6H, H-OC*H*$_3$); 3.78-3.59 (m, 2H, C*H*$_2$CH$_2$CN); 3.59-3.40 (m, 2H, NC*H*CH$_3$); 3.38-3.24 (m, 2H, H-5'); 2.75, 2.65 (2t, 2H, J = 5.9 Hz, CH$_2$C*H*$_2$CN); 2.55-2.43 (m, 1H, H-2'α); 2.24-2.14 (m, 1H, H-2'β); 1.131 (d, J = 6.8 Hz), 1.109 (d, J = 6.3 Hz), 1.096 (d, J = 6.5 Hz), 0.995 (d, J = 6.8 Hz) (12H, NCHC*H*$_3$).

^{13}C{^{1}H} NMR (125.7 MHz, d_6-DMSO) δ 160.9 (C2); 158.2 (2C, C4'''); 144.589, 144.573 (C1''); 140.0 (C4); 135.29, 135.27, 135.18, 135.14 (2C, C1'''); 133.2, 133.0 (C6); 129.8-129.7 (4C, C2'''); 127.9 (br, 2C, C3''); 127.72, 127.68 (2C, C2''); 126.8 (br, C4''); 119.64, 119.60 (C3); 118.9, 118.8 (*C*N); 113.2 (br, 4C, C3'''); 105.49, 105.44 (C5); 86.02, 85.96 (*C*Ar$_3$); 84.8-84.3 (2C, C1',4'); 72.6 (d, $^2J_{C,P}$ = 17.8 Hz), 72.0 (d, $^2J_{C,P}$ = 16.4 Hz) (C3'); 62.7, 62.5 (C5'); 58.4 (d, $^2J_{C,P}$ = 18.8 Hz), 58.3 (d, $^2J_{C,P}$ = 18.3 Hz) (*C*H$_2$CH$_2$CN); 55.07, 55.05 (2C, O*C*H$_3$); 42.6 (d, 2C, $^2J_{C,P}$ = 12.4 Hz, N*C*HCH$_3$); 40.0, 39.8 (C2'); 24.37, 24.31, 24.28, 24.25, 24.23, 24.17 (4C, NCH*C*H$_3$); 19.81 (d, $^3J_{C,P}$ = 7.2 Hz), 19.78 (d, $^3J_{C,P}$ = 7.0 Hz) (CH$_2$*C*H$_2$CN).

$^{31}P\{^1H\}$ NMR (202.4 MHz, d_6-DMSO, ref. extn. 85% H_3PO_4) δ 149.0, 148.6 (1:1 ratio).

ESI-MS: Calcd. for $[M+Na]^+$: 736.3128, Found: 736.3106.

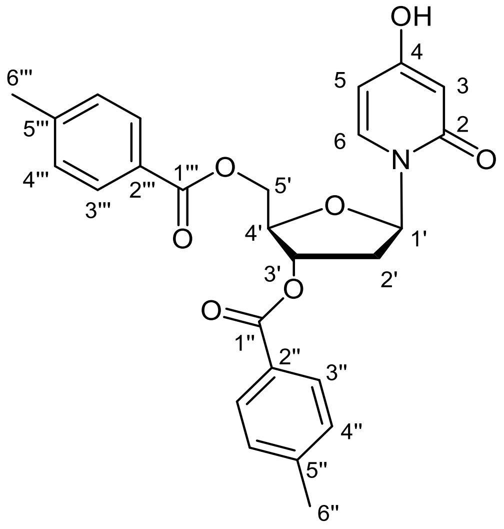

3',5'-Di-*O*-(p-toluoyl)-2'-deoxy-3-deazauridine (2b) was prepared by silyl-Hilbert-Johnson reaction with modification described earlier.[8] In a round-bottom two-neck flask (see **Figure S1** below) was placed dry $CHCl_3$ (20 mL) and distillation started with a speed 1-1.1 mL/min. Fresh chloroform was added through dropping funnel with the same speed to keep the reaction volume (20 mL) constant. Silylated pyrimidine (obtained by refluxing of 2,4-dihydroxypyridine (**1b**, 2.22 g, 20 mmol) in HMDS (9.6 ml, 46 mmol) in the presence of catalytic amount of $(NH_4)_2SO_4$ for 1 hr followed by evaporation of volatiles *in vacuo*) and Hoffer's chlorosugar (5.18 g, 13.3 mmol) were subsequently added to the boiling $CHCl_3$. TMSCl produced in the reaction was distilled off as an azeotrope with $CHCl_3$. Distillation was continued for 15 min keeping reaction volume constant. Reaction mixture was diluted with CH_2Cl_2 (80 mL) followed by addition of EtOH (5 ml) and H_2O (0.5 ml). Solvents were rotary evaporated *in vacuo* and residue was crystallised from EtOH (250 ml). Pure β-anomer **2b** was obtained in 50% yield (3.90 g) after second recrystallisation from EtOH.

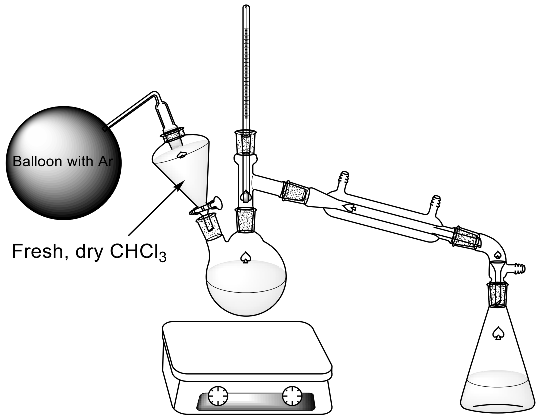

Figure S1. Set-up used for the synthesis of **2b**.

^{1}H NMR (500 MHz, d_6-DMSO) δ 10.82 (br. s, 1H, H-O*H*), 7.91 (d, 2H, $J_{3'',4''}$ = 8.2 Hz, H-3''), 7.86 (d, 2H, $J_{3''',4'''}$ = 8.2 Hz, H-3'''), 7.59 (d, 1H, $J_{5,6}$ = 7.8 Hz, H-6), 7.34 (d, 2H, $J_{3'',4''}$ = 8.2 Hz, H-4''), 7.31 (d, 2H, $J_{3''',4'''}$ = 8.2 Hz, H-4'''), 6.48 (dd, 1H, $J_{1',2'\beta}$ = 8.0 Hz, $J_{1',2'\alpha}$ = 6.0 Hz, H-1'), 5.87 (dd, 1H, $J_{5,6}$ = 7.8 Hz, $^4J_{3,5}$ = 2.6 Hz, H-5), 5.60 (d, 1H, $^4J_{3,5}$ = 2.6 Hz, H-3), 5.58 (m, 1H, H-3'), 4.65-4.55 (m, 2H, H-5'), 4.54-4.49 (m, 1H, H-4'), 2.60 (ddd, 1H, $J_{2'\alpha,2'\beta}$ = 14.4 Hz, $J_{1',2'\alpha}$ = 6.0 Hz, $J_{2'\alpha,3'}$ = 2.4 Hz, H-2'α), 2.40 (m, 1H, H-2'β), 2.38 (s, 3H, H-6''), 2.36 (s, 3H, H-6''').

^{13}C{^{1}H} NMR (125.7 MHz, d_6-DMSO) δ 166.8 (C4); 165.6 (C1'''); 165.3 (C1''); 162.7 (C2); 144.1, 144.0 (2C, C5'',5'''); 134.1 (C6); 129.49, 129.40, 129.38, 129.34 (8C, C3'',4'',3''',4'''); 126.6, 126.5 (2C, C2'',2'''); 100.7 (C5); 98.1 (C3); 84.3 (C1'); 81.6 (C4'); 75.0 (C3'); 64.3 (C5'); 37.5 (C2'); 21.24, 21.21 (2C, C6'',6''').

ESI-MS: Calcd. for $[M+H]^+$: 464.1709, Found: 464.1703.

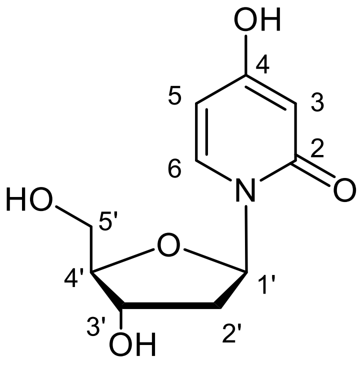

2'-Deoxy-3-deazauridine (3b).

3',5'-Di-*O*-(p-toluoyl)-2'-deoxy-3-deazauridine (**2b**, 4.64 g, 10 mmol) was suspended in MeOH (370 mL) and aq. ammonia (28%, 37 mL) was added and left overnight at room temperature. TLC analysis (CH_2Cl_2/acetone, 80:20) indicated the presence of the starting material. More aq. ammonia (28%, 37 mL) was added and reaction mixture was stirred at 45 °C for 4 days. Reaction mixture was rotary evaporated *in vacuo*, co-evaporated with H_2O (2 × 25 mL), dissolved in H_2O (50 mL) and filtered. Deprotected nucleoside was freeze-dried and used in the next step without further purification. The procedure results in quantitative yield of **3b** with p-toluoylamide as a major impurity.

Analytical sample of **3b** was obtained when the residue, after freeze-drying, was dissolved in MeOH and Et_2O was added. Precipitate formed was filtered out and filtrate was kept overnight at -20 °C providing crystalline **3b**.

^{1}H NMR (500 MHz, d_6-DMSO) δ 7.74 (d, 1H, $J_{5,6}$ = 7.7 Hz, H-6), 6.33 (dd, 1H, $J_{1',2'\beta}$ = 7.5 Hz, $J_{1',2'\alpha}$ = 6.0 Hz, H-1'), 5.91 (dd, 1H, $J_{5,6}$ = 7.7 Hz, $^4J_{3,5}$ = 2.6 Hz, H-5), 5.54 (d, 1H, $^4J_{3,5}$ = 2.6 Hz, H-3), 5.23, 5.00 (2br. s, 2H, 3',5'-O*H*), 4.24-4.19 (m, 1H, H-3'), 3.81-3.77 (m, 1H, H-4'), 3.61-3.52 (m, 2H, H-5'), 2.15 (ddd, 1H, $J_{2'\alpha,2'\beta}$ = 13.2 Hz, $J_{1',2'\alpha}$ = 6.0 Hz, $J_{2'\alpha,3'}$ = 3.2 Hz, H-2'α), 1.96-1.88 (m, 1H, H-2'β).

^{13}C{^{1}H} NMR (125.7 MHz, d_6-DMSO) δ 166.7 (C4), 162.8 (C2), 134.5 (C6), 100.3 (C5), 97.9 (C3), 87.4 (C4'), 83.8 (C1'), 70.5 (C3'), 61.4 (C5'), 40.9 (C2').

ESI-MS: Calcd. for $[M+H]^+$: 228.0872, Found: 228.0867.

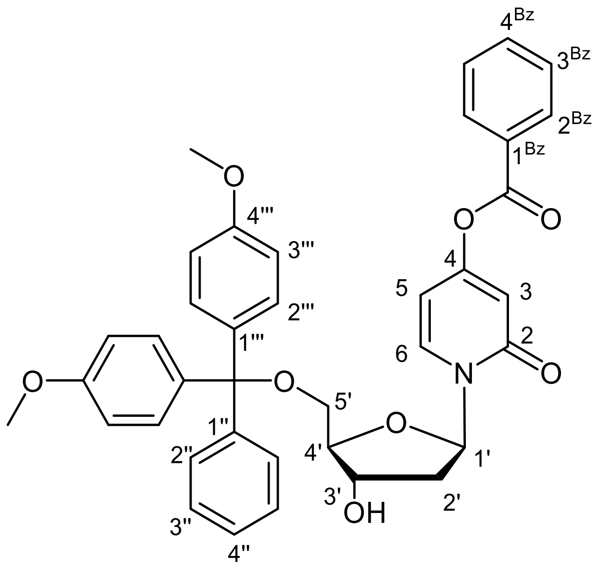

5'-*O*-(4,4'-Dimethoxytrityl)-4-*O*-benzoyl-2'-deoxy-3-deazauridine (4b). The deprotected nucleoside **3b** (1.76 g, 7.7 mmol) was co-evaporated twice with abs. pyridine (25 mL), dissolved in abs. pyridine (80 mL), cooled to 0°C and 4,4'-dimethoxytritylchloride (2.87 g, 8.5 mmol) was added. Reaction was stirred overnight on a melting ice bath. TLC analysis (CH_2Cl_2/EtOH, 95:5) showed clean formation of DMT-protected nucleoside. Reaction mixture was cooled to 0 °C, benzoic anhydride (1.92 g, 8.5 mmol) was added and reaction mixture was stirred overnight on a melting ice bath. TLC analysis (CH_2Cl_2/EtOH, 95:5) showed complete disappearance of the DMT-protected nucleoside and formation of a faster moving product. MeOH (1mL) was added to quench excess of reagents and after 10 min, the reaction mixture was evaporated *in vacuo.* Flash chromatography on silica eluting with a step gradient of EtOH (0→10%) in CH_2Cl_2 afforded **4b** (3.3 g, 81% on **2b**). According to ^{1}H NMR analysis, compound contains ca. 20% mol (5% w) p-toluoylamide.

^{1}H NMR (500 MHz, d_6-DMSO) δ 8.11-8.08 (m, 2H, H-2^{Bz}), 7.87 (d, 1H, $J_{5,6}$ = 7.7 Hz, H-6), 7.78-7.73 (m, 1H, H-4^{Bz}), 7.63-7.58 (m, 2H, H-3^{Bz}), 7.42-7.38 (m, 2H, H-2''), 7.35-7.30 (m, 2H, H-3''), 7.29-7.25 (m, 4H, H-2'''), 7.25-7.20 (m, 1H, H-4''), 6.93-6.88 (m, 4H, H-3'''), 6.38 (d, 1H, $J_{3,5}$ = 2.5 Hz, H-3), 6.36 (t, 1H, J = 6.2 Hz, H-1'), 6.21 (dd, 1H, $J_{5,6}$ = 7.7 Hz, $J_{3,5}$ = 2.5 Hz, H-5), 5.40 (d, 1H, $J_{3',OH}$ = 4.6 Hz, H-O*H*), 4.35-4.29 (m, 1H, H-3'), 4.02-3.98 (m, 1H, H-4'), 3.729, 3.726 (2s, 6H, H-C*H*$_3$), 3.32-3.24 (m, 2H, H-5'), 2.38 (ddd, 1H, $J_{2'\alpha,2'\beta}$ = 13.4 Hz, J = 6.4 Hz, J = 4.6 Hz, H-2'α), 2.11 (dt, 1H, $J_{2'\alpha,2'\beta}$ = 13.4 Hz, J = 6.4 Hz, H-2'β).

^{13}C{^{1}H} NMR (125.7 MHz, d_6-DMSO) δ 163.2 (*C*OPh); 161.8 (C2); 160.0 (C4); 158.17, 158.15 (2C, C4'''); 144.6 (C1''); 135.5, 135.3 (2C, C1'''); 134.5 (C4^{Bz}); 134.3 (C6); 130.0 (2C, C2^{Bz}); 129.8, 129.7 (4C, C2'''); 129.1 (2C, C3^{Bz}); 128.3 (C1^{Bz}); 128.0 (2C, C3''); 127.8 (2C, C2''); 126.8 (C4''); 113.3 (4C, C3'''); 109.4 (C3); 102.3 (C5); 85.9 (*C*Ar$_3$); 85.8 (C4'); 84.8 (C1'); 70.0 (C3'); 63.2 (C5'); 55.05 (2C, *C*H$_3$); 41.05 (C2').

ESI-MS: Calcd. for $[M+Na]^+$: 656.2261, Found: 656.2255.

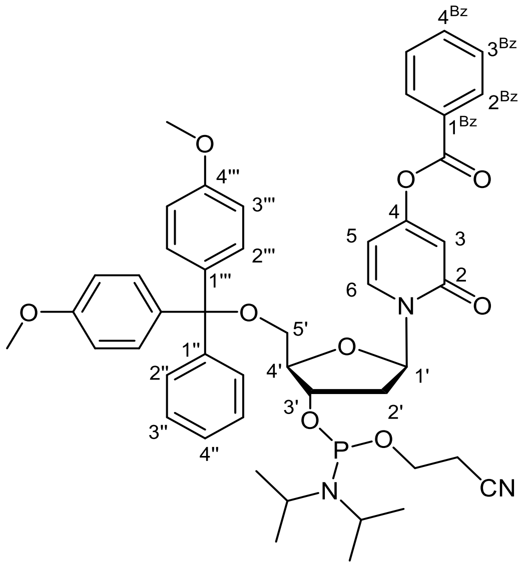

5'-*O*-(4,4'-Dimethoxytrityl)-3'-*O*-(*N*,*N*-di*iso*propylamino-2-cyanoethoxyphosphanyl)-4-*O*-benzoyl-2'-deoxy-3-deazauridine (5b). 5'-*O*-DMT-2'-deoxy-4-*O*-benzoyl-3-deazauridine (**4b**, 1.9 g, 3.0 mmol) was co-evaporated with abs. CH_2Cl_2 (50 mL), dissolved in abs. CH_2Cl_2 (60 mL) and Et_3N (0.84 mL, 6.0 mmol) followed by *N*,*N*-di*iso*propylamino-2-cyanoethoxychlorophosphine (1.20 g, 5.1 mmol) were added under argon. After stirring for 60 min reaction was complete by TLC analysis (CH_2Cl_2/acetone/Et_3N, 90:5:5). Reaction mixture was diluted with CH_2Cl_2 (150 mL), washed with satd. $NaHCO_3$ (3 × 300 mL), brine (300 mL), filtered through Na_2SO_4 and rotary evaporated *in vacuo*. The residue was purified by flash chromatography on silica (packed in CH_2Cl_2/Et_3N, 90:10 and washed with CH_2Cl_2/EtOAc, 95:5) eluting with a step gradient of AcOEt (5→10 %) in CH_2Cl_2. Fractions with the product were collected, rotary evaporated *in vacuo*, to yield **5b** as a mixture of two diastereomers (2.1 g, 84%).

^{1}H NMR (500 MHz, d_6-DMSO) δ 8.12-8.07 (m, 2H, H-2^{Bz}); 7.91 (d, $J_{5,6}$ = 7.8 Hz), 7.88 (d, $J_{5,6}$ = 7.7 Hz) (1H, H-6); 7.78-7.72 (m, 1H, H-4^{Bz}); 7.63-7.57 (m, 2H, H-3^{Bz}); 7.42-7.37 (m, 2H, H-2''); 7.35-7.20 (m, 7H, H-3'',4'',2'''); 6.92-6.86 (m, 4H, H-3'''); 6.42-6.32 (m, 2H, H-3,1'); 6.27-6.22 (m, 1H, H-5); 4.60-4.50 (m, 1H, H-3'); 4.16 (q, J = 3.8 Hz), 4.11 (q, J = 4.2 Hz) (1H, H-4'); 3.82-3.59 (m, 2H, CH_2CH$_2$CN); 3.728, 3.726, 3.723, 3.721 (4s, 6H, H-OCH_3); 3.60-3.42 (m, 2H, NCHCH$_3$); 3.37-3.28 (m, 2H, H-5'); 2.76, 2.66 (2t, 2H, J = 5.9 Hz, CH$_2$CH_2CN); 2.60-2.45 (m, 1H, H-2'α); 2.31-2.20 (m, 1H, H-2'β); 1.136, 1.114, 1.005 (3d, J = 6.7 Hz), 1.110 (d, J = 6.8 Hz) (12H, NCHCH_3).

^{13}C{^{1}H} NMR (125.7 MHz, d_6-DMSO) δ 163.2 (*C*OPh); 161.8 (C2); 160.114, 160.105 (C4); 158.22, 158.21 (2C, C4'''); 144.5 (C1''); 135.36, 135.34, 135.20, 135.16 (2C, C1'''); 134.6-134.2 (2C, C6,4^{Bz}); 130.0 (2C, C2^{Bz}); 129.83, 129.80, 129.76, 129.74 (4C, C2'''); 129.1 (2C, C3^{Bz}); 128.25 (C1^{Bz}); 127.95, 127.94 (2C, C3''); 127.76, 127.71 (2C, C2''); 126.9 (C4''); 119.0, 118.8 (CN); 113.3 (4C, C3'''); 109.48, 109.46 (C3), 102.48, 102.42 (C5); 86.08, 86.01 (*C*Ar$_3$); 85.05-84.45 (2C, C1',4'); 72.7 (d, $^2J_{C,P}$ = 18.1 Hz), 72.2 (d, $^2J_{C,P}$ = 16.0 Hz) (C3'); 62.8, 62.6 (br, C5'); 58.4 (d, $^2J_{C,P}$ = 18.7 Hz), 58.3 (d, $^2J_{C,P}$ = 18.6 Hz) (*C*H$_2$CH$_2$CN); 55.06, 55.05 (2C, O*C*H$_3$); 42.7 (d, 2C, $^2J_{C,P}$ = 12.6 Hz, N*C*HCH$_3$); 39.9, 39.8 (C2'); 24.40, 24.34, 24.31, 24.28, 24.25, 24.20 (4C, NCH*C*H$_3$); 19.85 (d, $^3J_{C,P}$ = 7.0 Hz), 19.81 (d, $^3J_{C,P}$ = 7.1 Hz) (CH$_2$*C*H$_2$CN).

^{31}P{^{1}H} NMR (202.4 MHz, d_6-DMSO, extn. ref. 85% H_3PO_4) δ 149.0, 148.7 (1:1 ratio).

ESI-MS: Calcd. for $[M+Na]^+$: 856.3339, Found: 856.3324.

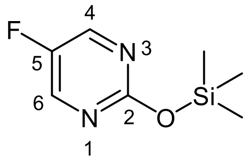

5-Fluoro-2-trimethylsilyloxypyrimidine was prepared from 5-fluoro-2-hydroxypyrimidine (**1c**, 2.34 g, 20 mmol) by refluxing in HMDS (7.6 mL, 36 mmol) in the presence of catalytic amount of $(NH_4)_2SO_4$ for 4 hr (until all solids dissolved). The mixture was distilled *in vacuo* on a water aspirator, providing the desired product (bp 100-101 °C at 40 mbar, 3.41 g, 89%).

^{1}H NMR (500.1 MHz, $CDCl_3$) δ 8.31 (s, 2H, C*H*), 0.36 (s, 9H, C*H₃*).

^{13}C{^{1}H} NMR (125.7 MHz, $CDCl_3$) δ 160.0 (d, $^4J_{C,F}$ = 1.9 Hz, *C*O), 154.3 (d, $^1J_{C,F}$ = 253.6 Hz, *C*F), 146.6 (d, 2C, $^2J_{C,F}$ = 22.4 Hz, *C*H), 0.0 (s, 3C, *C*H_3).

^{19}F NMR (376.5 MHz, $CDCl_3$, extn. ref. $CFCl_3$ (neat)) δ -150.6.

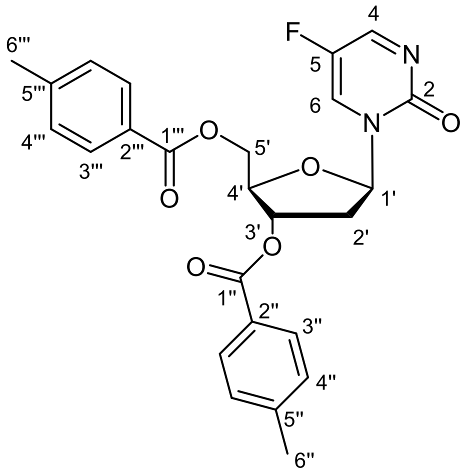

3',5'-Di-*O*-(p-toluoyl)-2'-deoxy-5-fluorozebularine (2c) To the solution of 5-fluoro-2-trimethylsilyloxypyrimidine (2.28 g, 12.3 mmol) in dichloroethane (60 mL) freshly distilled $SnCl_4$ (4.8 g, 18.4 mmol) and Hoffer's chlorosugar (2.38 g, 6.1 mmol) were sequentially added at -35 °C . Reaction mixture was stirred for 1.5 h at -35 °C, pyridine (10 mL) and H_2O (50 mL) were added and reaction mixture was stirred at rt for 1h followed by addition of H_2O (150 mL). The resulting mixture was extracted with CH_2Cl_2 (4 × 200 mL), combined organic layers were filtered through Na_2SO_4, rotary evaporated *in vacuo* and co-evaporated with toluene (50 mL). The residue was purified by flash chromatography on silica eluting with a step gradient of acetone (0→30 %) in CH_2Cl_2. Fractions containing nucleosides **2c** were concentrated and ^{1}H NMR analysis showed that this was a mixture of anomers (2.48 g, 87%, β/α = 9:1). Pure β-anomer (1.3 g, 45%) was isolated after second chromatography separation with a slow stepwise gradient of acetone (0→20 %) in CH_2Cl_2. R_f 0.31 (α-anomer), 0.41 (β-anomer) (acetone/CH_2Cl_2, 5/95).

^{1}H NMR (500 MHz, d_6-DMSO) δ 8.81 (d, 1H, $^4J_{4,6}$ = 3.8 Hz, H-4), 8.39 (dd, 1H, $^4J_{4,6}$ = 3.8 Hz, $J_{H6,F}$ = 4.3 Hz, H-6), 7.94-7.90 (m, 2H, H-3''), 7.83-7.79 (m, 2H, H-3'''), 7.37-7.33 (m, 2H, H-4''), 7.31-7.27 (m, 2H, H-4'''), 6.20-6.14 (m, 1H, H-1'), 5.61-5.56 (m, 1H, H-3'), 4.70-4.63 (m, 3H, H-4',5'), 2.82 (ddd, 1H, $J_{2'\alpha,2'\beta}$ = 14.7 Hz, *J* = 6.3 Hz, *J* = 2.8 Hz, H-2'α), 2.60-2.52 (m, 1H, H-2'β), 2.39 (s, 3H, H-6''), 2.37 (s, 3H, H-6''').

$^{13}C\{^1H\}$ NMR (125.7 MHz, d_6-DMSO) δ 165.5 (C1'''); 165.2 (C1''); 157.7 (d, $^2J_{C4,F}$ = 23.2 Hz, C4); 153.0 (C2); 145.3 (d, $J_{C5,F}$ = 236.9 Hz, C5); 144.1 (C5''); 144.0 (C5'''); 130.2 (d, $^2J_{C6,F}$ = 33.8 Hz, C6); 129.5 (2C, C3''); 129.34, 129.33 (4C, C4'',4'''); 129.2 (2C, C3'''); 126.47, 126.43 (2C, C2'',2'''); 88.0 (C1'); 82.8 (C4'); 74.6 (C3'); 64.1 (C5'); 37.7 (C2'); 21.21, 21.16 (2C, C6'',6''').

^{19}F NMR (376.5 MHz, d_6-DMSO, extn. ref. $CFCl_3$ (neat)) δ -160.46 (d, $J_{H6,F}$ = 4.3 Hz).

ESI-MS: Calcd. for $[M+Na]^+$: 489.1438, Found: 489.1427.

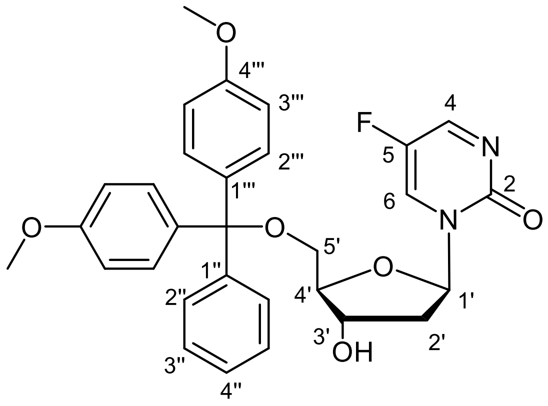

5'-*O*-(4,4'-Dimethoxytrityl)-2'-deoxy-5-fluorozebularine (4c). 3',5'-Ditoluoyl protected 2'-deoxy-5-fluorozebularine (**2c**, 3.85 g, 8.25 mmol) was dissolved in satd. ammonia solution in MeOH (~7 M solution, 450 mL). Reaction mixture was stirred at room temperature for 48 h, rotary evaporated *in vacuo*, co-evaporated with H_2O and freeze-dried from H_2O to result in 2'-deoxy-5-fluorozebularine (**3c**) that was used in the next step without further purification.

An analytical sample of the nucleoside was isolated from 20 mg of the mixture using preparative TLC (20 × 20 cm, elution system MeOH/CH_2Cl_2, 10:90, two times). According to NMR analysis, the nucleoside exists as a pair of isomers – "open" and "cyclic", therefore two sets of signals are present in ^1H and ^{13}C spectra. Designations "open" and "cyclic" are used to distinguish between the isomers. Integral intensities of the signals are reported mostly as fractional numbers that reflect the ratio of isomers.

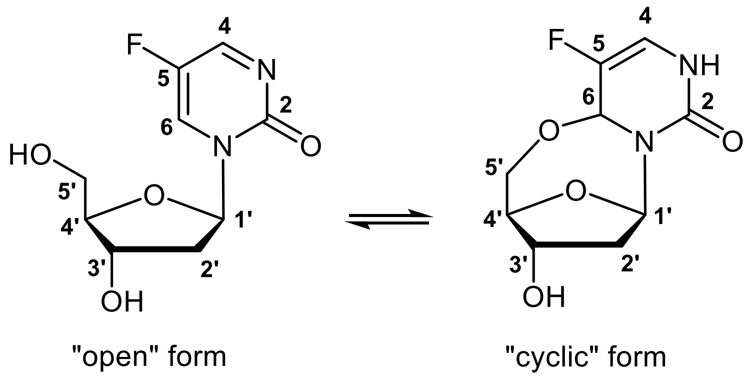

^1H NMR (400 MHz, d_6-DMSO) δ 8.79 (d, 0.6H, $^4J_{4,6}$ = 3.9 Hz, H-4-open), 8.69 (dd, 1H, $^4J_{4,6}$ = 3.9 Hz, $J_{H,F}$ = 4.6 Hz, H-6-open, N*H*-cyclic), 6.49 (dd, 0.4H, J = 6.9 Hz, J = 5.4 Hz, H-4-cyclic), 6.02-

5.96 (m, 0.6H, H-1'-open), 5.76-5.72 (m, 0.4H, H-1'-cyclic), 5.65 (d, 0.4H, J = 4.1 Hz, H-6-cyclic), 5.45-5.00 (3br. s, 1.6H, OH), 4.28-4.13 (m, 1.4H, H-3'-open, H-3',4'-cyclic), 3.91-3.85 (m, 0.6H, H-4'-open), 3.79 (d, 0.4H, ^2J = 12.5 Hz, H-5'-cyclic), 3.74-3.54 (m, 1.6H, H-5'-open, H-5'-cyclic), 2.40-2.29 (m, 1H, H-2'α-open, H-2'α-cyclic), 2.14-2.04 (m, 1H, H-2'β-open, H-2'β-cyclic).

^{13}C{^{1}H} NMR (100.6 MHz, d_6-DMSO) δ 157.0 (d, $^2J_{C4,F}$ = 23.0 Hz, C4-open), 153.1 (C2-open), 148.7 (d, $^4J_{C2,F}$ = 1.4 Hz, C2-cyclic), 145.3 (d, $J_{C5,F}$ = 235.4 Hz, C5-open), 138.9 (d, $J_{C5,F}$ = 231.1 Hz, C5-cyclic), 130.5 (d, $^2J_{C6,F}$ = 34.3 Hz, C6-open), 110.3 (d, $^2J_{C4,F}$ = 34.2 Hz, C4-cyclic), 90.2 (C4'-cyclic), 88.1 (C4'-open), 87.9 (C1'-cyclic), 87.1 (C1'-open), 82.4 (d, $^2J_{C6,F}$ = 34.4 Hz, C6-cyclic), 71.3 (C3'-cyclic), 70.7 (C5'-cyclic), 69.0 (C3'-open), 60.2 (C5'-open), 45.9 (C2'-cyclic), 40.9 (C2'-open).

2'-Deoxy-5-fluorozebularine (**3c**) was co-evaporated with abs. pyridine (50 mL) dissolved in abs. pyridine (80 mL), cooled to 0 °C and 4,4'-dimethoxytritylchloride (4.17 g, 12.3 mmol) was added. Reaction was stirred overnight at room temperature. Consumption of the starting nucleoside **3c** as well as formation of the DMT-protected **4c** was monitored by ^{1}H NMR: 200 µL reaction mixture was mixed with 400 µL d_6-DMSO and ^{1}H spectra were recorded after 3.5 h and overnight. Total disappearance of both signals of H6 protons at 8.69 ppm (H6-open) and 5.65 ppm (H6-cyclic) as well as synchronous appearance of H6 signal of DMT-protected compound at 8.28 ppm proves reversible equilibrium between "open" and "cyclic" forms of the nucleoside **3c**. The reaction mixture was evaporated *in vacuo*. The residue was dissolved in CH_2Cl_2 (400 mL), washed with H_2O (400 mL), satd. $NaHCO_3$ (400 mL), filtered through Na_2SO_4 and concentrated *in vacuo*. Flash chromatography on silica (packed in CH_2Cl_2/Et_3N, 99:1 and washed with CH_2Cl_2) eluting with a step gradient of acetone (0→40%) in CH_2Cl_2 afforded **4c** (2.65 g, 60% over two steps).

^{1}H NMR (700 MHz, d_6-DMSO) δ 8.79 (d, 1H, J = 3.8 Hz, H-4), 8.28 (app. t, 1H, J = 4.0 Hz, H-6), 7.40-7.36 (m, 2H, H-2''), 7.33-7.29 (m, 2H, H-3''), 7.29-7.24 (m, 4H, H-2'''), 7.25-7.20 (m, 1H, H-4''), 6.91-6.87 (m, 4H, H-3'''), 6.03-5.98 (m, 1H, H-1'), 5.40 (d, 1H, $J_{OH,3'}$ = 4.8 Hz, OH), 4.34-4.29 (m, 1H, H-3'), 4.02 (app. dt, 1H, J = 4.9 Hz, $J_{4',5'}$ = 2.9 Hz, H-4'), 3.73 (s, 6H, OCH_3), 3.32 (dd, 1H, ^2J = 10.8 Hz, $J_{4',5'}$ = 5.1 Hz, H-5'), 3.23 (dd, 1H, ^2J = 10.8 Hz, $J_{4',5'}$ = 2.9 Hz, H-5'), 2.42 (ddd, 1H, $^2J_{2'\alpha,2'\beta}$ = 13.6 Hz, J = 6.6 Hz, J = 5.2 Hz, H-2'α), 2.22 (ddd, 1H, $^2J_{2'\alpha,2'\beta}$ = 13.6 Hz, J = 6.5 Hz, J = 5.3 Hz, H-2'β).

^{13}C{^{1}H} NMR (176.1 MHz, d_6-DMSO) δ 158.14, 158.13 (2C, C4'''); 157.2 (d, $^2J_{C4,F}$ = 22.9 Hz, C4); 152.9 (C2); 145.2 (d, $J_{C5,F}$ = 237.1 Hz, C5); 144.7 (C1''); 135.3, 135.2 (2C, ;C1''') 129.8 (d, $^2J_{C6,F}$ = 33.4 Hz, C6); 129.7 (4C, C2'''); 127.90 (2C, C3''); 127.6 (2C, C2''); 126.8 (C4''); 113.27, 113.25 (4C, C3'''); 87.2 (C1'), 86.2 (C4'); 86.0 (CAr$_3$); 69.3 (C3'); 62.9 (C5'); 50. 03, 50.02 (2C, OCH$_3$), 40.6 (C2').

ESI-MS: Calcd. for $[M+Na]^+$: 555.1907, Found: 555.1905.

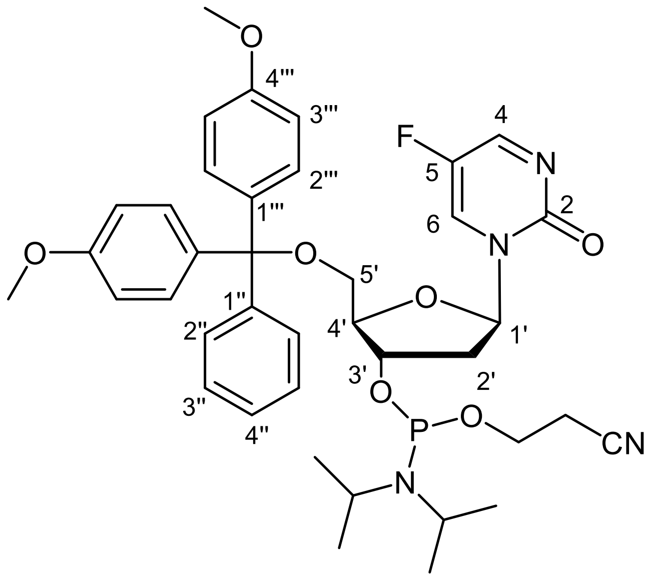

5'-*O*-(4,4'-Dimethoxytrityl)-3'-*O*-(*N*,*N*-di*iso*propylamino-2-cyanoethoxyphosphanyl)-2'-deoxy-5-fluorozebularine (5c). 5'-*O*-DMT-2'-deoxy-5-fluorozebularine (**4c**, 2.6 g, 4.85 mmol) was co-evaporated with abs. CH_2Cl_2 (50 mL), dissolved in abs. CH_2Cl_2 (100 mL) and Et_3N (1.3 mL, 9.6 mmol) followed by *N*,*N*-di*iso*propylamino-2-cyanoethoxychlorophosphine (1.7 g, 7.3 mmol) were added under argon. After stirring for 30 min reaction was complete by TLC analysis (CH_2Cl_2/EtOAc, 7:3, plate pretreated with Et_3N/Hex 1:9 and dried). Reaction mixture was diluted with CH_2Cl_2 (50 mL), washed with satd. $NaHCO_3$ (3 × 100 mL), filtered through Na_2SO_4 and rotary evaporated *in vacuo*. The residue was purified by flash chromatography on silica (packed in toluene/Et_3N, 99:1) eluting with step gradients of acetone (0→15 %) and Et_3N (1→4 %) in toluene. Fractions with the product were collected, rotary evaporated *in vacuo*, to yield **5c** as a mixture of two diastereomers (3.1 g, 89%).

^{1}H NMR (700.1 MHz, d_6-DMSO): δ 8.802, 8.801 (2d, 1H, $J_{4,6}$ = 3.8 Hz, H-4); 8.34-8.30 (m, 1H, H-6); 7.42-7.37 (m, 2H, H-2''); 7.33-7.20 (m, 7H, H-3'',4'',2'''); 6.91-6.85 (m, 4H, H-3'''); 6.04, 6.00 (2m, 1H, H-1'); 4.60-4.49 (m, 1H, H-3'); 4.17, 4.12 (2m, 1H, H-4'); 3.74, 3.73 (2s, 6H, OCH_3); 3.72-3.60 (m, 2H, CH_2CH$_2$CN); 3.60-3.47 (m, 2H, CHCH$_3$); 3.40-3.26 (m, 2H, H-5'); 2.76 (t, J = 5.9 Hz), 2.65 (t, J = 6.0 Hz) (2H, CH$_2$CH_2CN); 2.61-2.51 (m, 1H, H-2'α); 2.43-2.33 (m, 1H, H-2'β); 1.14, 1.110, 1.109, 1.00 (4d, 12H, J = 6.8 Hz, CHCH_3).

^{13}C{^{1}H} NMR (176.1 MHz, d_6-DMSO): δ 158.17, 158.16 (2C, C4'''); 157.44 (d, $^2J_{C4,F}$ = 22.8 Hz); 157.38 (d, $^2J_{C4,F}$ = 22.5 Hz) (C4); 152.89, 152.87 (C2); 145.2 (d, $^1J_{C5,F}$ = 236.9 Hz, C5); 144.5 (C1''); 135.19, 135.18, 135.06, 135.04 (2C, C1'''); 130.0, 129.8 (d, $^2J_{C6,F}$ = 33.5 Hz, C6); 129.70, 129.68 (4C, C2'''); 127.87, 127.86 (2C, C3'''); 127.57, 127.55 (2C, C2''); 126.79, 126.77 (C4''); 118.90, 118.75 (*C*N); 113.2 (4C, C3'''); 87.07, 87.13 (C1'); 86.1, 86.0 (*C*Ar$_3$); 85.2 (d, $^3J_{C4',P}$ = 4.5 Hz), 84.9 (d, $^3J_{C4',P}$ = 5.9 Hz) (C4'); 71.8 (d, $^2J_{C3',P}$ = 17.3 Hz), 71.3 (d, $^2J_{C3',P}$ = 15.9 Hz) (C3'); 62.4, 62.1 (C5'); 58.35 (d, $^2J_{C,P}$ = 19.0 Hz), 58.23 (d, $^2J_{C,P}$ = 18.8 Hz) (*C*H$_2$CH$_2$CN); 55.03, 55.02, 55.01, 55.00 (2C, O*C*H$_3$); 42.62, 42.61 (2d, $^2J_{C,P}$ = 12.4 Hz, 2C, *C*HCH$_3$); 39.6, 39.3 (C2', in DEPT); 24.34, 24.27, 24.24, 24.20 (4d, $^3J_{C,P}$ = 7 Hz, 4C, CH*C*H$_3$); 19.81 (d, $^3J_{C,P}$ = 7.1 Hz), 19.78 (d, $^3J_{C,P}$ = 7.0 Hz) (CH$_2$*C*H$_2$CN).

^{19}F NMR (376.4 MHz, d_6-DMSO, extn. ref. $CFCl_3$ (neat)): δ -160.49, -160.47 (2d, J = 4.1 Hz).

^{31}P{^{1}H} NMR (202.4 MHz, d_6-DMSO, extn. ref. 85% H_3PO_4) δ 149.1, 148.8 (1:1 ratio).

ESI-MS: Calcd. for $[M+Na]^+$: 755.2986, Found: 755.2982.

2. Synthesis of modified oligodeoxynucleotides (oligos)

Oligos were prepared on a MerMade-4 DNA/RNA synthesizer (BioAutomation) on a 5 µmol scale using standard manufacturer's protocol. Modified phosphoramidites **5a-c** were used in the synthesis of modified oligonucleotide sequences (**Table S1**). Coupling time for modified phosphoramidites was increased to 5 min. The final detritylation step was omitted and DMT-ON oligos containing **3dadZ** and **3dadU** were cleaved from the solid support and deprotected with 28% ammonia solution (1.0 mL) at 60 °C overnight. After filtering, an aq. solution of 0.3 M $LiClO_4$ (0.5 mL) was added and oligonucleotides were precipitated with acetone (14 mL). **5FdZ**-containing oligos were deprotected on the solid support by a two-step procedure with 10% Et_2NH in acetonitrile for 5 min, followed by incubation of the support in ethylenediamine/toluene mixture (1/1, v/v) for 2 hrs at room temperature.[9] The support was washed with toluene (3 × mL), dried *in vacuo* and the deprotected **5FdZ**-containing oligo was released in H_2O (1 mL).

The DMT-ON oligonucleotides were isolated by reversed-phase HPLC on 250/10 mm, 5 µm, 300 Å C18 column (Phenomenex) in a linear gradient of CH_3CN (0→60% over 15 min, 4.6 mL/min) in 0.1 M TEAA buffer (pH 7.0) with a detection at 260 nm. DMT-ON oligonucleotides were freeze-dried and manually detritylated with 80% aq. AcOH (2 mL) during 20 min at room temperature. 3M AcONa solution (0.5 mL) was then added and oligonucleotides were precipitated with 2-propanol (11 mL). DMT-OFF oligonucleotides were purified by RP-HPLC on 250/4.6 mm, 5 µm, 300 Å C18 column (Phenomenex) in a gradient of CH_3CN (0→25% for 20 min, 1.3 mL/min) in 0.1 M TEAA buffer (pH 7.0). Fractions containing desired oligonucleotides were combined, freeze-dried, dissolved in milli-Q water (1.5 mL) and desalted on a NAP-25 column (GE Healthcare) against 'saltless buffer' consisting of 10 µM Tris-HCl (pH 8.0), 1 µM EDTA and 0.001% w/v NaN_3. Pure oligos were quantified by measuring absorbance at 260 nm, analysed by ESI-MS in negative mode using 10 µM aq. oligo solution supplemented with 20% MeOH (Table S1) and concentrated by freeze-drying.

Table S1. Modified oligos synthesised and purified using protocols described above

**Name**	**DNA sequence 5'→3'**	**ESI-MS, [M] found/calcd**
**3dadZ-oligo**	ATTT-**3dadZ**-ATTT	2661.6 / 2661.5
**3dadU-oligo**	ATTT-**3dadU**-ATTT	2678.1 / 2677.5
**5FdZ-oligo**	ATTT-**5FdZ**-ATTT	2680.6 / 2680.5
**CC5FdZ-oligo**	ATTCC-**5FdZ**-AATT	2964.8 / 2963.5
**T4-5FdZ-oligo**	TTTT-**5FdZ**-AT	2063.3 / 2063.4

Instrument:ANALYTICAL_500loop Sequence:MVK_DNA Page 1 of 1

Chromatogram

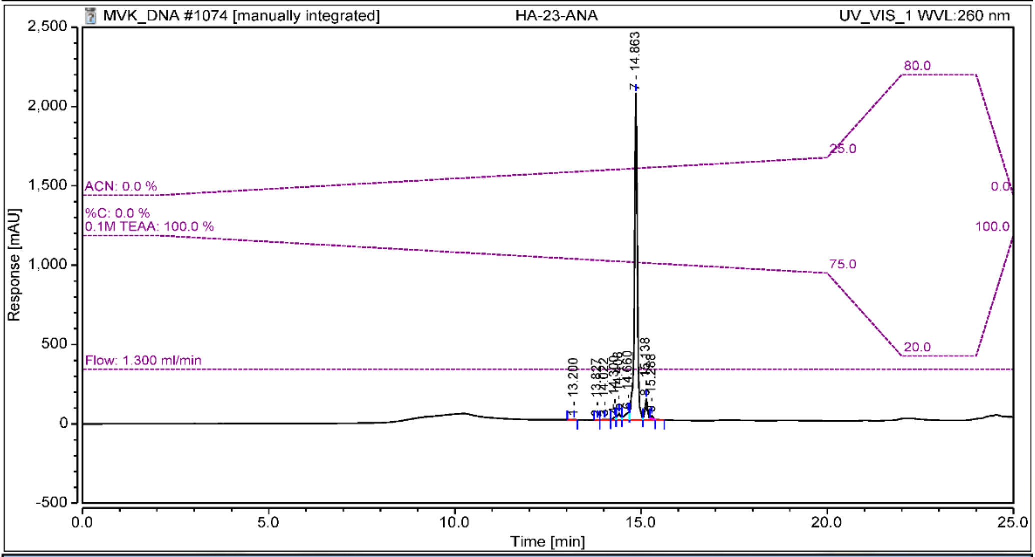

Integration Results

No.	Peak Name	Retention Time min	Area mAU*min	Height mAU	Relative Area %	Relative Height %	Amount
n.a.	Component 1	n.a.	n.a.	n.a.	n.a.	n.a.	n.a.
1		13.200	0.173	1.444	0.08	0.06	n.a.
2		13.827	0.147	1.757	0.07	0.08	n.a.
3		14.022	0.348	2.319	0.17	0.10	n.a.
4		14.300	1.402	16.837	0.68	0.73	n.a.
5		14.408	4.099	40.269	1.98	1.74	n.a.
6		14.660	6.507	47.657	3.14	2.06	n.a.
7		14.863	180.883	2056.698	87.30	88.79	n.a.
8		15.138	12.447	131.221	6.01	5.66	n.a.
9		15.288	1.190	18.241	0.57	0.79	n.a.
**Total:**			**207.195**	**2316.442**	**100.00**	**100.00**	

test/Integration

Chromeleon (c) Dionex
Version 7.2.8.10783

Chart S1. RP-HPLC profile of **5FdZ-oligo**. Note that the broad peak at 10 min is an artefact of the column.

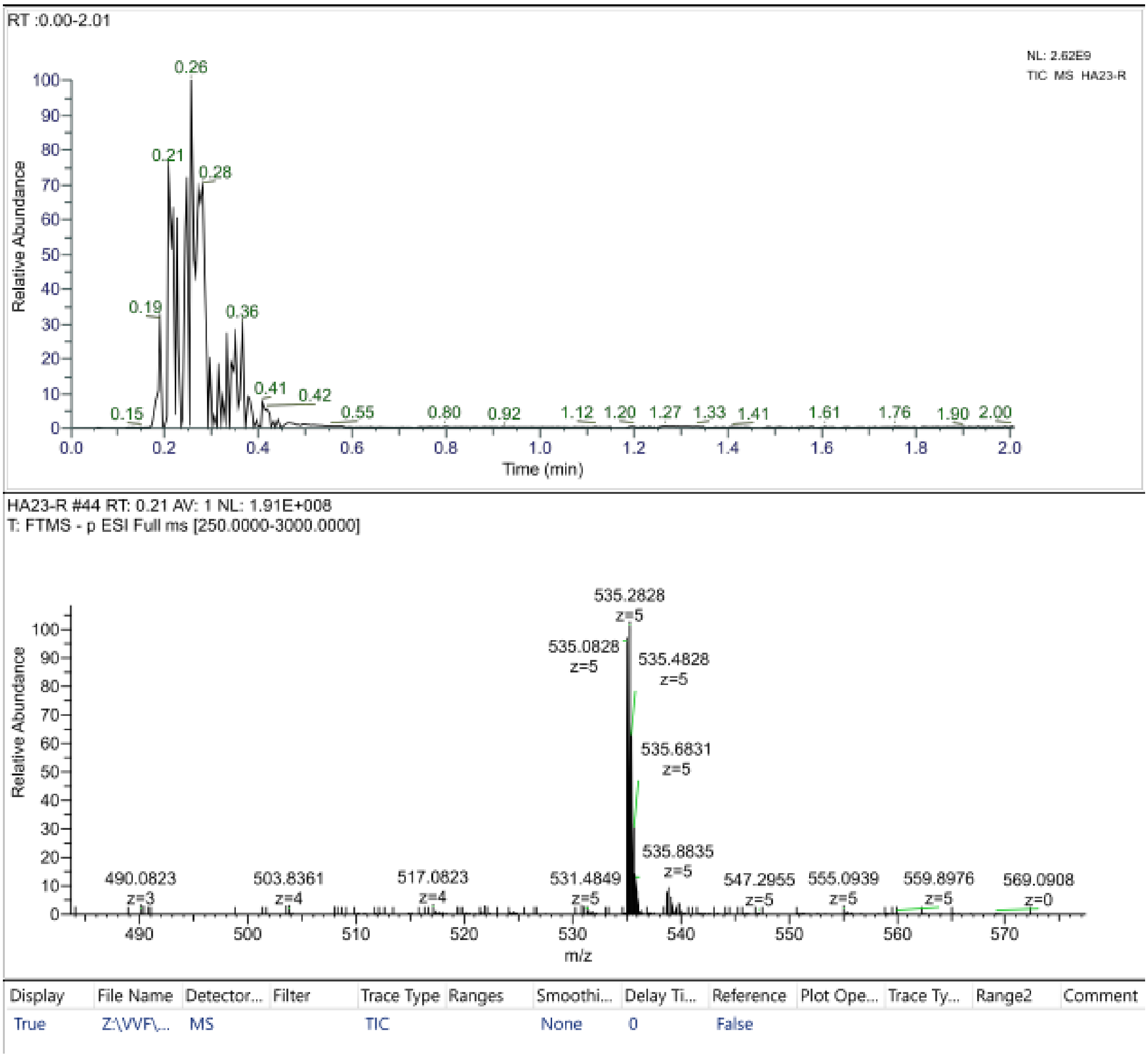

Chart S2. Mass-spectrometry data for **5FdZ-oligo.** The top graph is a chromatogram of a mass-spec sample, bottom graph is a mass-spec data: m/z = 535.0828 (charge -5), which corresponds to 2680.6 Da.

Instrument:ANALYTICAL_500loop Sequence:MVK_DNA

Chromatogram

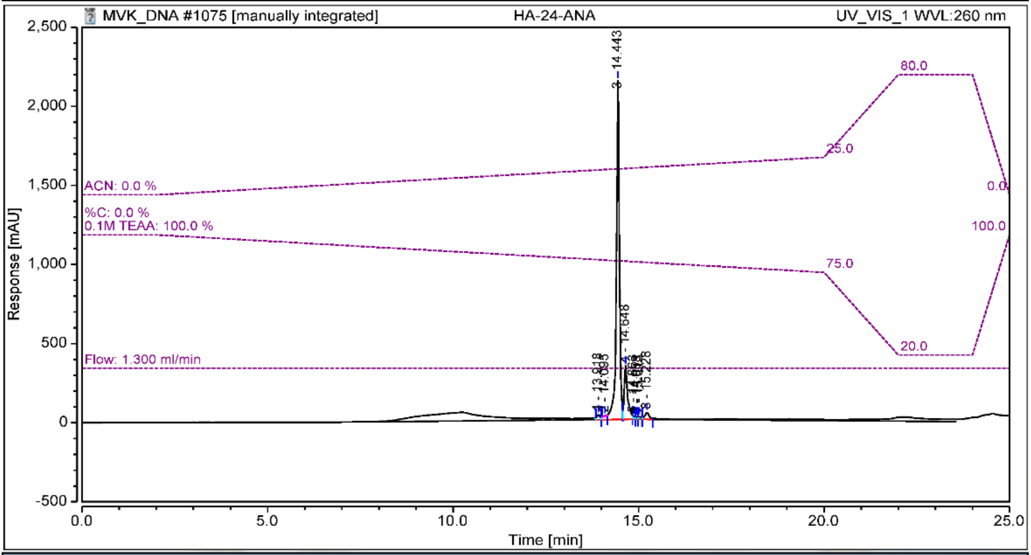

Integration Results

No.	Peak Name	Retention Time min	Area mAU*min	Height mAU	Relative Area %	Relative Height %	Amount
n.a.	Component 1	n.a.	n.a.	n.a.	n.a.	n.a.	n.a.
1		13.918	3.242	32.851	1.28	1.26	n.a.
2		14.095	0.249	3.590	0.10	0.14	n.a.
3		14.443	204.854	2141.220	80.83	82.00	n.a.
4		14.648	34.835	337.871	13.74	12.94	n.a.
5		14.863	1.586	20.766	0.63	0.80	n.a.
6		14.958	0.900	14.675	0.35	0.56	n.a.
7		15.013	1.607	16.741	0.63	0.64	n.a.
8		15.228	6.170	43.562	2.43	1.67	n.a.
**Total:**			**253.442**	**2611.277**	**100.00**	**100.00**	

test/Integration

Chromeleon (c) Dionex
Version 7.2.8.10783

Chart S3. RP-HPLC profile of **CC5FdZ-oligo**. Note that the broad peak at 10 min is an artefact of the column.

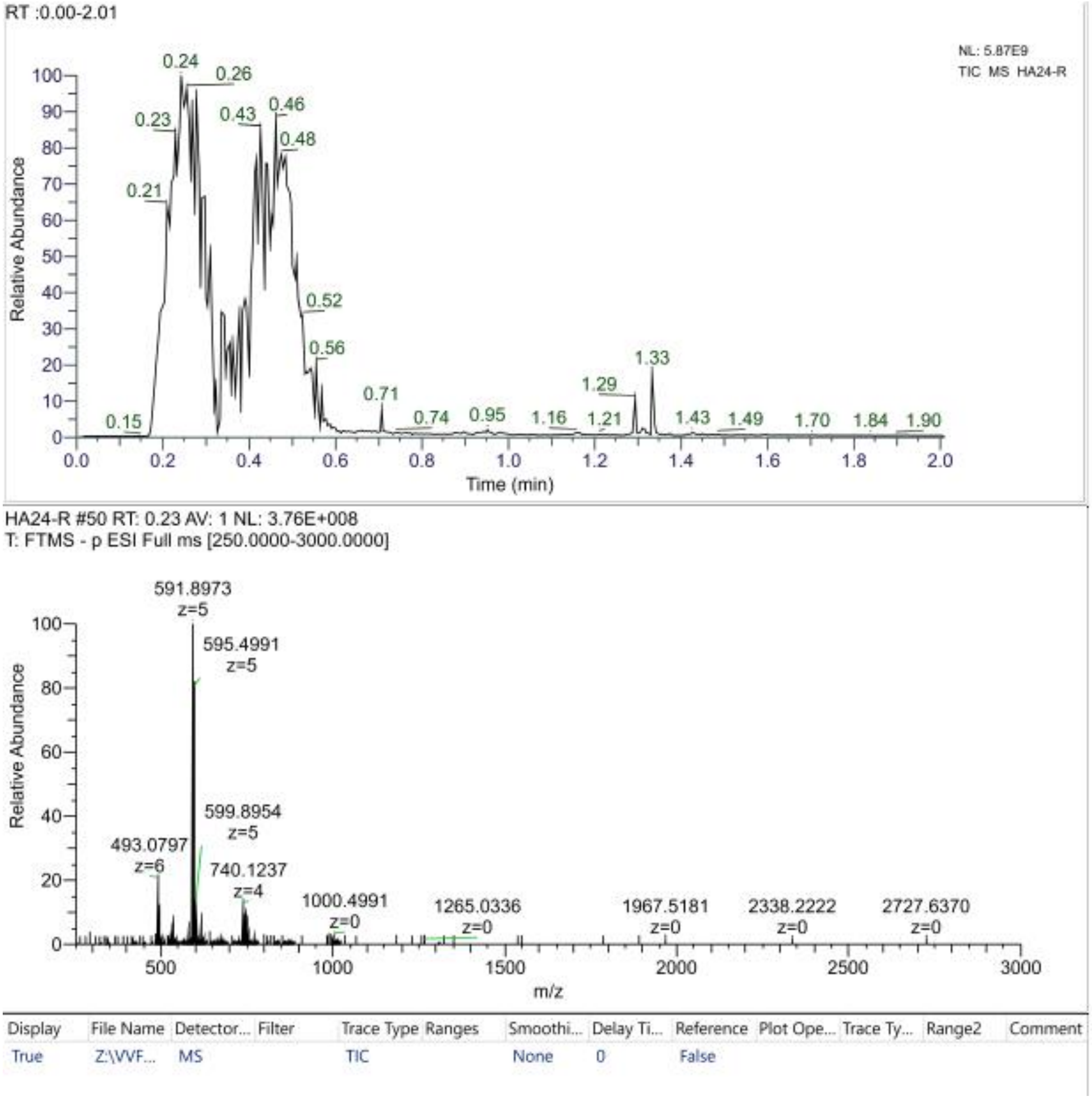

Chart S4. Mass-spectrometry data for **CC5FdZ-oligo.** The top graph is a chromatogram of a mass-spec sample, bottom graph is a mass-spec data: m/z = 591.8973 (charge -5), which corresponds to 2964.8 Da.

Chromatogram and Results

Injection Details

Injection Name:	**HA-33 ANA**	*Run Time (min):*	**25.00**
Vial Number:	**GE1**	*Injection Volume:*	**10.00**
Injection Type:	**Unknown**	*Channel:*	**UV_VIS_1**
Calibration Level:		*Wavelength:*	**260**
Instrument Method:		*Bandwidth:*	**2**
Processing Method:	**3D Qualitative**	*Dilution Factor:*	**1.0000**
Injection Date/Time:	**08/Jun/18 11:14**	*Sample Weight:*	**1.0000**

Chromatogram

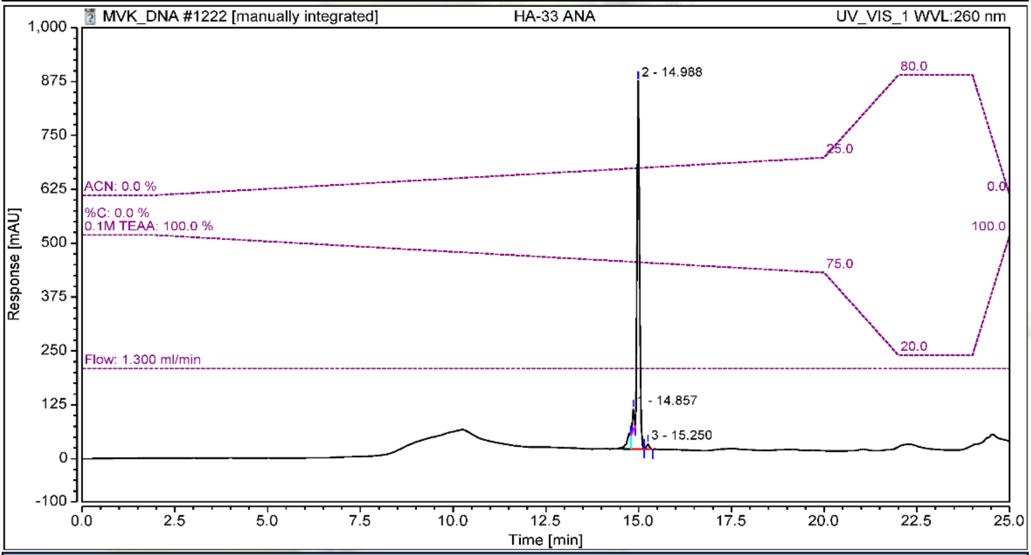

Integration Results

No.	Peak Name	Retention Time min	Area mAU*min	Height mAU	Relative Area %	Relative Height %	Amount n.a.
1		14.857	2.680	48.389	3.49	5.29	n.a.
2		14.988	72.998	853.918	95.18	93.40	n.a.
3		15.250	1.019	11.975	1.33	1.31	n.a.
**Total:**			**76.697**	**914.282**	**100.00**	**100.00**	

test/Integration

Chromeleon (c) Dionex
Version 7.2.8.10783

Chart S5. RP-HPLC profile of **T4-5FdZ-oligo**. Note that the broad peak at 10 min is an artefact of the column.

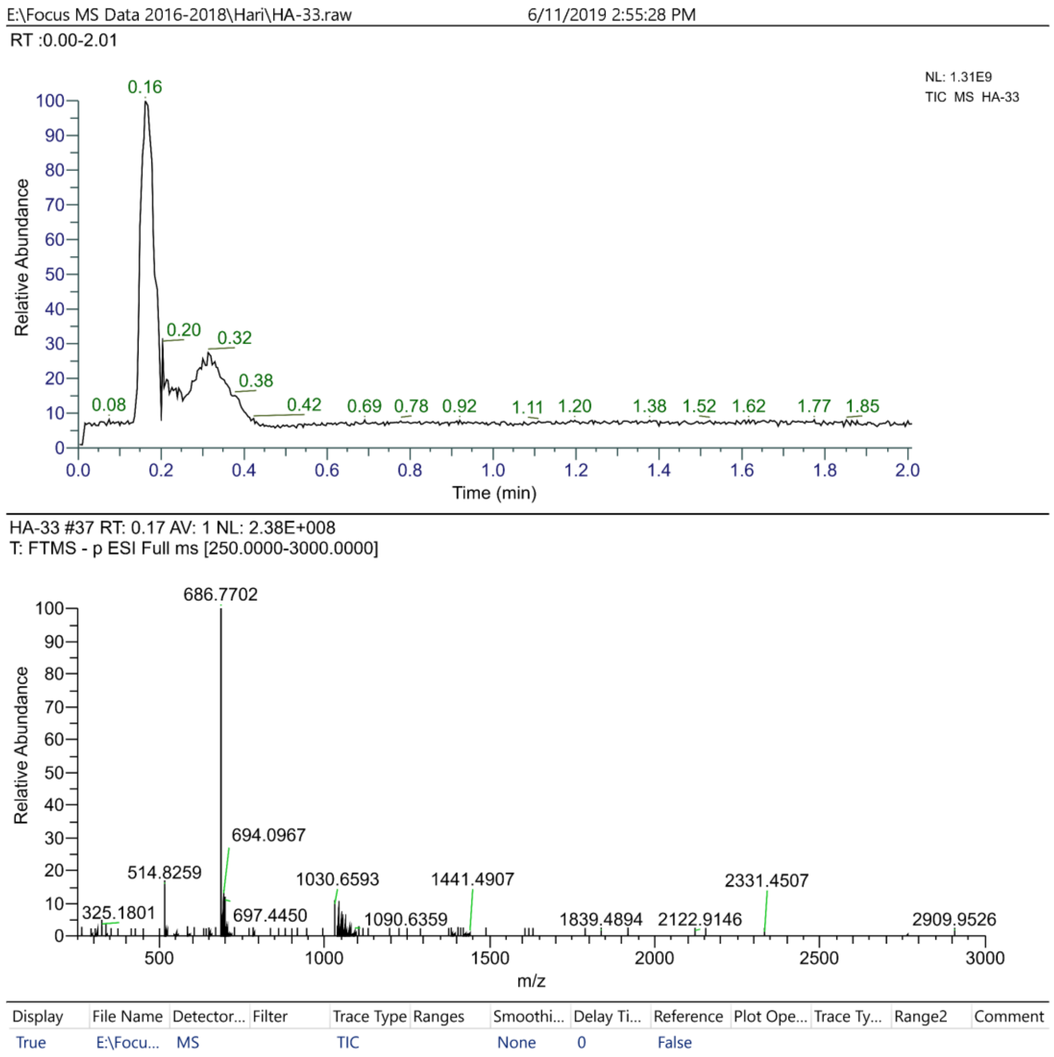

Chart S6. Mass-spectrometry data for **T4-5FdZ-oligo.** The top graph is a chromatogram of a mass-spec sample, bottom graph is a mass-spec data: m/z = 686.7702 (charge -3), which corresponds to 2063.3 Da.

3. Protein expression and purification

Proteins were prepared as described previously[10] (**Figure S2**). In short, the A3B C-terminal domain (residues 187 to 378, UniProt ID Q9UH17) was cloned into pET24a vector (Novagen) to produce A3B$_{CTD}$ proteins with a non-cleavable C-terminal His$_6$-tag (LEHHHHHH), adapted from a previously described study.[11] Derivative construct previously reported was used in this study, A3B$_{CTD}$ -QM-ΔL3-AL1swap expressed in the *E. coli* strain C41(DE3)pLysS (Lucigen). *E. coli* culture was grown at 37 °C in LB medium. Once mid-log growth phase was established the culture was supplemented with 100 µM zinc chloride, before inducing protein expression by the addition of *iso*propyl β-D-1-thiogalactopyranoside (IPTG) at a final concentration of 0.5 µM and incubating overnight at 18 °C.

GST-fused A3G$_{CTD}$(191-384, NM_021822, wt) was purified as previously described.[12] Briefly, the N-terminal glutathione S-transferase (GST)-fused A3G$_{CTD}$ was expressed in *E. coli* BL21(DE3) cells overnight at 17 °C. After harvesting, the cells were resuspended in 50 mM sodium phosphate buffer (pH 7.4) and lysed by sonication. After ultracentrifugation at 25,000 *g* for 10 min, the supernatant was added to glutathione (GSH)-Sepharose, which was subsequently washed. For kinetic analysis, the GST fusion protein was eluted from the Sepharose matrix with 100 mM GSH in phosphate buffer. By using filtration at 4,000 *g*, the buffer was changed to a solution containing 75 mM sodium phosphate and 75 mM citrate, at pH 5.5.

Full length A3G was produced from HEK293-6E cells grown planktonically following transient transfection as described previously[13] using a pTT5 protein expression plasmid containing GST-tagged full length A3G. Following expression and lysis, using sonication and M-PER™, the protein was solubilised in 50 mM Tris-HCl, 150 mM NaCl, 10% glycerol, pH 7.5. Next, the full-length A3G was purified using column chromatography with a GST-trap FF column. Following the GST-trap column run the GST-tag was cleaved using PreScission Protease and the post-cleavage reaction material was re-run on the column. Afterwards, size exclusion chromatography was carried out in 50 mM Tris-HCl, 150 mM NaCl, 10% glycerol, 1 mM DTT, pH 7.5 using a Superose 12 column. The full length A3G peak was concentrated and frozen and stored at -80 °C until required for testing.

```
A3GCTD      ------EILRHSMDPPTFTFNFNNEPWVRGRHETYLCYEVERMHNDTWVLLNQRRGFLCN
A3A         MEASPASGPRHLMDPHIFTSNFNNG---IGRHKTYLCYEVERLDNGTSVKMDQHRGFLHN
A3BCTD      ------EILRYLMDPDTFTFNFNNDPLVLRRRQTYLCYEVERLDNGTWVLMDQHMGFLCN
A3B-AL1     ------EILRYLMDPDTFTSNFNNG---IGRHKTYLCYEVERLDNGTSVKMDQHMGFLCN
                     *. ***  ** ****         *********. * * * ..*  *** *

A3GCTD      QAPHKHGFLEGRHAELCFLDVIPFWKLDLDQDYRVTCFTSWSPCFS--CAQEMAKFISKN
A3A         QAKNLLCGFYGRHAELRFLDLVPSLQLDPAQIYRVTWFISWSPCFSWGCAGEVRAFLQEN
A3BCTD      EAKNLLCGFYGRHAELRFLDLVPSLQLDPAQIYRVTWFISWSPCFSWGCAGEVRAFLQEN
A3B-AL1     E--------SGRHAELRFLDLVPSLQLDPAQIYRVTWFISWSPCFSWGCAGEVRAFLQEN
                      ****** ***..*   **  * **** * *******  ** *   *.  *

A3GCTD      KHVSLCIFTARIYDDQGRCQEGLRTLAEAGAKISIMTYSEFKHCWDTFVDHQGCPFQPW
A3A         THVRLRIFAARIYDYDPLYKEALQMLRDAGAQVSIMTYDEFKHCWDTFVDHQGCPFQPW
A3BCTD      THVRLRIFAARIYDYDPLYKEALQMLRDAGAQVSIMTYDEFEYCWDTFVYRQGCPFQPW
A3B-AL1     THVRLRIKAARIYDYDPLYKEALQMLRDAGAQVSIMTYDEFEYCWDTFVYRQGCPFQPW
             ** * *  *****      *.*  *  *** .***** ** .******  ********

A3GCTD      DGLDEHSQDLSGRLRAILQNQEN
A3A         DGLDEHSQALSGRLRAILQNQGN
A3BCTD      DGLEEHSQALSGRLRAILQNQGN
A3B-AL1     DGLEEHSQALSGRLRAILQ----
            ***.**** **********
```

Figure S2. $A3B_{CTD}$ and $A3G_{CTD}$ are C-terminal domains of A3B and A3G. A3B-AL1 = $A3B_{CTD}$-QM-ΔL3-AL1 (Quadra Mutant: F200S, W228S, L230K, and F308K; loop3 deleted: Ala242 to Tyr250 replaced by Ser; loop 1 swapped with A3A). Green shaded are identical amino acids between A3A and $A3B_{CTD}$. Asterisks mark identical residues across the four constructs. Cyan shaded is I279 of A3B, corresponding Ile in A3A and Thr in the same position in A3G. I279 has contact with the target cytidine in A3B-AL1-DNA complex. Full-length A3G has an N-terminal domain in addition to the shown C-terminal domain (CTD) sequence.

A)

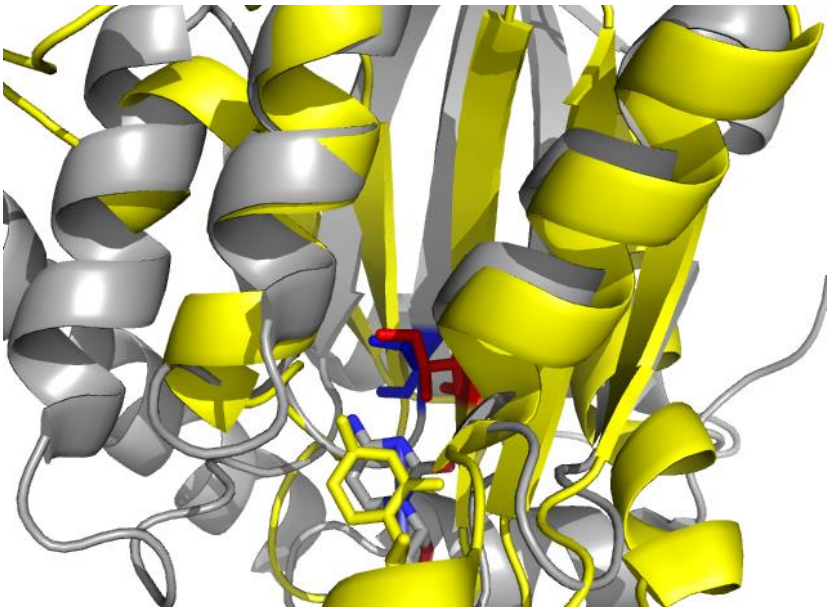

B)

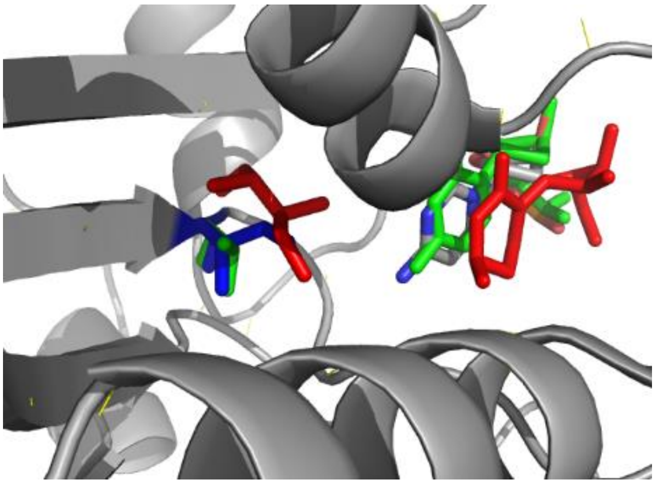

Figure S3. A) Overlay (RMSD = 0.675 Å, pymol (https://pymol.org)) of the active sites of the X-ray structures of A3B$_{CTD}$-AL1 (residues 253-298) containing ssDNA (grey, pdb-id: 5td5) and mouse CDA (yellow, pdb-id 2fr5, residues 65-110) in complex with tetrahydrouridine (shown is sticks). Target (0-position) 2'-deoxycytidine of ssDNA, Ile279 (red) and Ile87 (blue) are shown in sticks as well. The two protein sequences share only ~10% identity, but in the vicinity of the active site, conservation is high and alignment is close (A3B$_{CTD}$-AL1 [CDA]: His253 [Cys65], Cys284 [Cys99], Cys289 [Cys102] (Zn ligands); Glu255 [Glu67] (general acid-base)).

B) Overlay of the active sites of the X-ray structures of A3G$_{CTD}$ containing ssDNA (pdb-id: 6bux), A3B$_{CTD}$-AL1 (pdb-id: 5td5) and mouse CDA (pdb-id 2fr5). The A3G$_{CTD}$ is added to the overlay in Figure S3A (overlaid on A3B$_{CTD}$-AL1). Secondary structure elements of A3B$_{CTD}$-AL1 surrounding the active side are shown in grey. In sticks: Ile 87 and tetrahydrouridine of mouse CDA are shown in red; Thr 283 of A3G$_{CTD}$ and the target cytidine are shown in green. Ile 279 of A3B$_{CTD}$-AL1 is shown in blue and to highlight the position of the target cytidine, it is coloured according to its chemical composition (nitrogens are blue, carbons are grey).

4. Evaluation of inhibitors in NMR-based assay

For evaluation of inhibitors, a series of ^{1}H NMR spectra was recorded of the substrate 5'-ATTTCATTT at constant concentration of 350 µM in the presence of 50 nM of A3B$_{CTD}$-QM-ΔL3-AL1swap and constant concentrations of **3dadZ-**, **3dadU-**, or **5FdZ-oligos** (50 µM) in activity assay buffer (50 mM citrate-phosphate, 200 mM NaCl, 2 mM β-mercaptoethanol, 200 µM 4,4-dimethyl-4-silapentane-1-sulfonic acid (DSS); pH 5.5) containing 10 % deuterium oxide. Measurements were acquired on a 700-MHz Bruker NMR spectrometer equipped with a 1.7-mm cryoprobe at 298 K. The doublet signal of the H-5 proton of the cytosine (5.90 ppm, J = 8.5 Hz) was integrated and then converted to substrate concentration and plotted versus time of the reaction. This plot was then fitted with linear regression to determine the speed of the reaction in the presence of inhibitor at specific concentration, which is reported in Figure 3 in the main text. Determination of K_i for **5FdZ**-oligo was performed as previously described.[10] Briefly, a series of ^{1}H NMR spectra was recorded of the substrate 5'-ATTTCATTT at constant concentration of 350 µM with varying concentrations of the inhibitor 5'-ATTT**5FdZ**ATTT ranging from 5 µM to 100 µM in the presence of 50 nM of A3B$_{CTD}$-QM-ΔL3-AL1swap in activity assay buffer. After the speed of the reaction was determined at various inhibitor concentrations, the plot of inverse speed *versus* inhibitor concentration (Figure S4) was then fitted with linear regression (y = ax + b, a = 11.036 s×µM^{-2}; b = 63.848 s×µM^{-1}) to derive the inhibition constant (K_i) using the following formula

$$K_i = \frac{bK_m}{a(K_m + [S])}$$

and K_m of 197 µM and k_{cat} of 0.28 s^{-1} values obtained previously for A3B$_{CTD}$ -QM-ΔL3-AL1swap.[10] Uncertainty of K_i was calculated using error-propagation method.

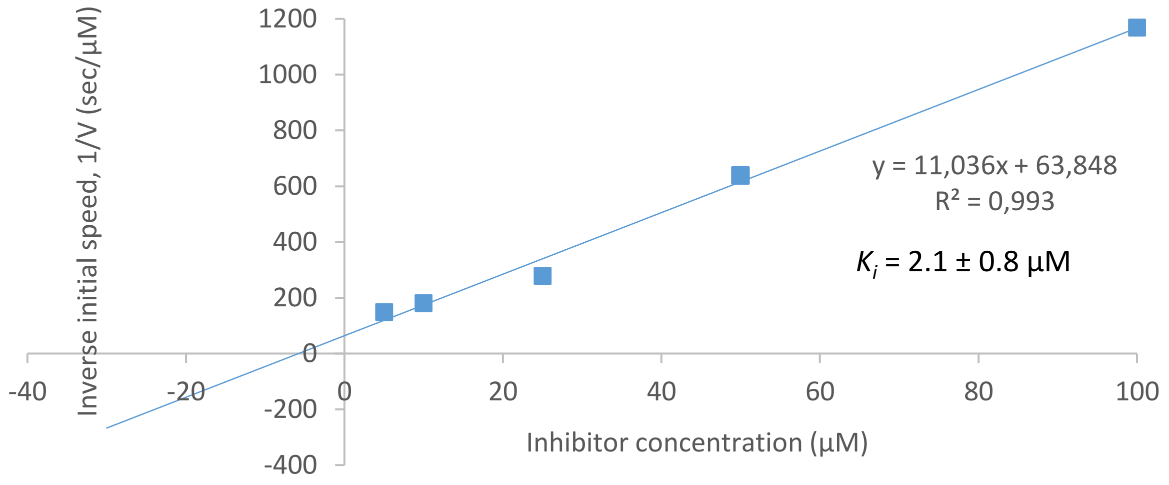

Figure S4. Inhibition of A3B$_{CTD}$-QM-ΔL3-AL1swap catalysed deamination of 5'-ATTTCATTT by **5FdZ**oligo.

Similarly, the inhibition constant for $A3G_{CTD}$ was calculated (Figure 4 in the main text): y = ax + b, a = 0.277 s×μM^{-2}; b = 31 s×μM^{-1}, K_m = 560 μM,[12] substrate concentration [s] = 342 μM).

5. Evaluation of inhibitors in fluorescence-based APOBEC3A ssDNA deamination assay and UDG inhibition control experiments

APOBEC deaminase activity assays were performed according to Li and colleagues with the following modifications.[14] Stock solutions of oligonucleotides in molecular biology grade water were serially diluted from 900 µM to 225 nM in protein dilution buffer (10 µL; 50 mM Tris-HCl, pH 7.5, 100 mM NaCl, 1 mM EDTA, 10% glycerol, and 0.5% Triton X-100) in black Nunc 384 flat bottom well plates (Thermo Scientific 262260). Recombinant (purified from HEK293T cells) full length A3A-mycHis (10 ng)[14] was delivered to each well in protein dilution buffer (10 µL) and incubated at 37 °C for 15 min. The deamination dual substrate oligo 5'-(6-FAM)-AAA-TAT-CCC-AAA-GAG-AGA-(TAMRA) (2 pmol) and uracil DNA glycosylase (UDG, 25 units) were then delivered in TE buffer (10 µL) and incubated at 37 °C for 30 min. Cleavage of oligonucleotides containing an abasic site is achieved using aqueous NaOH (4 M, 3 µl) and incubating at 37 °C for 30 min. The cleavage reaction was quenched using a neutralisation solution (40 µL; 2 M Tris-HCl, pH 7.9). Plates were read using a BioTek Synergy H1 plate reader with an excitation wavelength at 490 nm and emission at 520 nm. Each experiment was performed with three technical replicates per condition. Raw deaminase activity fluorescence values for individual concentrations were plotted and fit to GraphPad Prism (V. 7.0) log(inhibitor) vs. response – variable slope (four parameters) function yielding an IC_{50} for each biological replicate. Curve fits with R square values < 0.95 were discarded resulting in four biological replicates. The IC_{50} values for each biological replicate were then averaged and reported with their accompanying standard error of means (SEM) calculated in Microsoft Excel. The individual biological replicates are shown in Figure S5, panel A. To demonstrate that inhibition of A3A observed with **dZ**- and **5FdZ**-containing oligos was not due to an excess of ssDNA (compared to the fluorescent oligonucleotide assay substrate), this experiment was repeated using matched oligos 5'-TTTT**dU**AT and 5'-TTTT**C**AT in technical triplicate (Figure S5, panel B).

To ensure that oligonucleotide A3A inhibitors were not interfering with the second enzyme in the coupled assay, UDG, a separate fluorescence based assay was performed identically to that described above with the following modifications. Stock solutions of oligonucleotides in molecular biology grade water were diluted to 45 µM (final concentration 15 µM; this concentration was selected because complete A3A inhibition is observed by oligonucleotides in the previously described assay; see Figure S5, panel A) in protein dilution buffer (10 µL). Instead of A3A, UDG (25 units) was delivered to each well in protein dilution buffer (10 µL). Then, the UDG specific dual substrate oligo 5'-(6-FAM)-AAA-TAT-C-dU-C-AAA-GAG-AGA-(TAMRA) (2 pmol) was delivered in TE buffer (10 µL). Fluorescence data were normalised to the no-protein low control. Mean UDG activity (%) and standard deviations were plotted using GraphPad Prism (V. 7.0). This assay was performed in biological duplicate with at least five technical replicates per condition (individual biological replicates are shown in Figure S5, panel C). As a control, the experiment was also performed using the matched oligos 5'-TTTT**dU**AT and 5'-TTTT**C**AT in biological duplicate and technical triplicate (Figure S5, panel D).

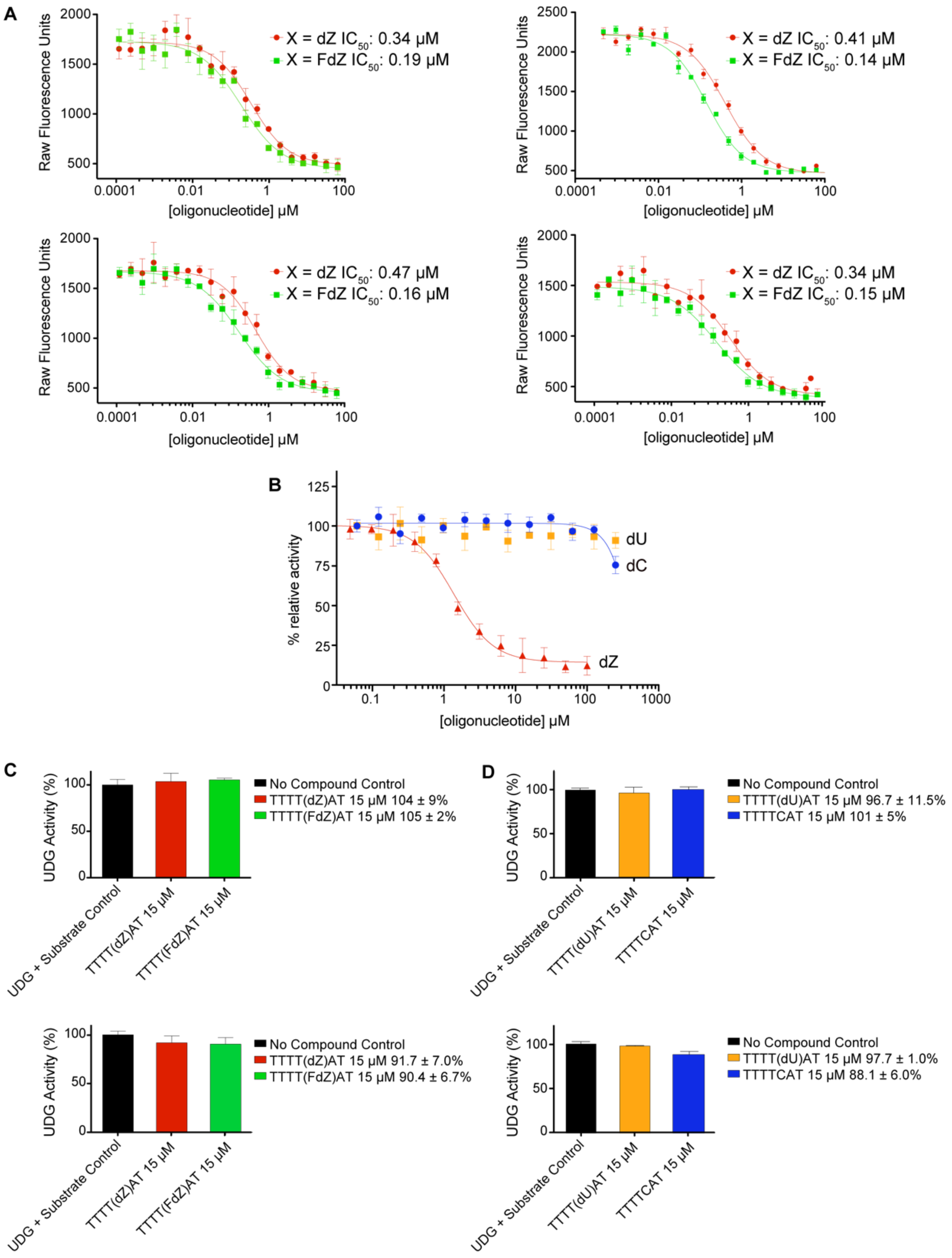

Figure S5. A) Individual biological replicates (with three technical replicates per condition) for the inhibition of human A3A deaminase activity by 5'-TTTT**X**AT (**X** = **dZ** *or* **5FdZ**). B) Control deaminase inhibition experiment using matched normal oligonucleotides 5'-TTTT**dU**AT and 5'-TTTT**C**AT, demonstrating that only the **dZ**/**5FdZ**-containing oligos inhibit hA3A at the concentrations tested. C) Control experiments to verify that 5'-TTTT**dZ**AT and 5'-TTTT**5FdZ**AT do not inhibit UDG-catalysed cleavage of uracil in ssDNA deaminase assay substrates. Data shown are individual biological replicates with at least five technical replicates per condition. D) Control experiments to verify that 5'-TTTT**dU**AT and 5'-TTTT**C**AT do not inhibit UDG-catalysed cleavage of uracil in ssDNA deaminase assay substrates. Data shown are individual biological replicates with at least three technical replicates per condition.

6. References:

[1] Kögler, M., Busson, R., De Jonghe, S., Rozenski, J., Van Belle, K., Louat, T., Munier-Lehmann, H., and Herdewijn, P. (2012) Synthesis and Evaluation of 6-Aza-2′-deoxyuridine Monophosphate Analogs as Inhibitors of Thymidylate Synthases, and as Substrates or Inhibitors of Thymidine Monophosphate Kinase in Mycobacterium tuberculosis, *Chem. Biodiv. 9*, 536-556.

[2] Gottlieb, H. E., Kotlyar, V., and Nudelman, A. (1997) NMR Chemical Shifts of Common Laboratory Solvents as Trace Impurities, *J. Org. Chem. 62*, 7512-7515.

[3] Chambers, M. C., Maclean, B., Burke, R., Amodei, D., Ruderman, D. L., Neumann, S., Gatto, L., Fischer, B., Pratt, B., Egertson, J., Hoff, K., Kessner, D., Tasman, N., Shulman, N., Frewen, B., Baker, T. A., Brusniak, M. Y., Paulse, C., Creasy, D., Flashner, L., Kani, K., Moulding, C., Seymour, S. L., Nuwaysir, L. M., Lefebvre, B., Kuhlmann, F., Roark, J., Rainer, P., Detlev, S., Hemenway, T., Huhmer, A., Langridge, J., Connolly, B., Chadick, T., Holly, K., Eckels, J., Deutsch, E. W., Moritz, R. L., Katz, J. E., Agus, D. B., MacCoss, M., Tabb, D. L., and Mallick, P. (2012) A cross-platform toolkit for mass spectrometry and proteomics, *Nat. Biotechnol. 30*, 918-920.

[4] www.mmass.org.

[5] Seela, F., and Bindig, U. (1989) Glycosylations of ambident anions of 2(1H)- and 4(1H)-pyridone and stereoselective synthesis of 2(1H)-pyrimidinone N-(2'-deoxy-α-D-ribofuranoside), *Liebigs Ann. Chem.*, 895-901.

[6] Durland, R. H., Rao, T. S., Revankar, R., Tinsley, J. H., Myrick, M. A., Seth, D. M., Rayford, J., Singh, P., and Jayaraman, K. (1994) Binding of T and T analogs to CG base pairs in antiparallel triplexes, *Nucleic Acids Res. 22*, 3233-3240.

[7] Urban, M., Joubert, N., Hocek, M., Alexander, R. E., and Kuchta, R. D. (2009) Herpes Simplex Virus-1 DNA Primase: A Remarkably Inaccurate yet Selective Polymerase, *Biochemistry 48*, 10866-10881.

[8] Kotick, M. P., Szantay, C., and Bardos, T. J. (1969) Synthesis of 5-s-substituted 2'-deoxyuridines. Study of the factors influencing the stereoselectivity of the silyl modification of the Hilbert-Johnson reaction, *J. Org. Chem. 34*, 3806-3813.

[9] (2013) Glen Research, Deprotection Supplement, Deprotection - Volumes 1-5.

[10] Kvach, M. V., Barzak, F. M., Harjes, S., Schares, H. A. M., Jameson, G. B., Ayoub, A. M., Moorthy, R., Aihara, H., Harris, R. S., Filichev, V. V., Harki, D. A., and Harjes, E. (2019) Inhibiting APOBEC3 activity with single-stranded DNA containing 2'-deoxyzebularine analogues, *Biochemistry 58*, 391-400.

[11] Shi, K., Carpenter, M. A., Banerjee, S., Shaban, N. M., Kurahashi, K., Salamango, D. J., McCann, J. L., Starrett, G. J., Duffy, J. V., Demir, O., Amaro, R. E., Harki, D. A., Harris, R. S., and Aihara, H. (2017) Structural basis for targeted DNA cytosine deamination and mutagenesis by APOBEC3A and APOBEC3B, *Nat Struct Mol Biol 24*, 131-139.

[12] Harjes, S., Solomon, W. C., Li, M., Chen, K.-M., Harjes, E., Harris, R. S., and Matsuo, H. (2013) Impact of H216 on the DNA binding and catalytic activities of the HIV restriction factor APOBEC3G, *J. Virol. 87*, 7008-7014.

[13] Durocher, Y., Perret, S., and Kamen, A. (2002) High-level and high-throughput recombinant protein production by transient transfection of suspension-growing human 293-EBNA1 cells, *Nucleic Acids Res 30*, E9.

[14] Li, M., Shandilya, S. M. D., Carpenter, M. A., Rathore, A., Brown, W. L., Perkins, A. L., Harki, D. A., Solberg, J., Hook, D. J., Pandey, K. K., Parniak, M. A., Johnson, J. R., Krogan, N. J., Somasundaran, M., Ali, A., Schiffer, C. A., and Harris, R. S. (2012) First-In-Class Small Molecule Inhibitors of the Single-Strand DNA Cytosine Deaminase APOBEC3G, *ACS Chem. Biol. 7*, 506-517.
